# MEMS Varifocal Optical Elements for Focus Control

**DOI:** 10.3390/mi16040482

**Published:** 2025-04-19

**Authors:** Chen Liu, Tong Wang, Xin Wang, Manpeng Chang, Yu Jian, Weimin Wang

**Affiliations:** 1 Key Laboratory of Optoelectronic Technology and Systems, Ministry of Education, Chongqing University, Chongqing 400044, China; 202308021013@stu.cqu.edu.cn (C.L.); 17784734256@163.com (T.W.); xinxinwang267@gmail.com (X.W.); cmp7756@163.com (M.C.); jianyu0221@163.com (Y.J.); 2 Defense Key Disciplines Laboratory of Novel Micro-Nano Devices and System Technology, Chongqing University, Chongqing 400044, China; 3 College of Optoelectronic Engineering, Chongqing University, Chongqing 400044, China

**Keywords:** MOEMS, focus control, varifocal mirror, tunable lens, micro-optical system

## Abstract

As microelectronic devices become more prevalent daily, miniaturization is emerging as a key trend, particularly in optical systems. Optical systems with volume scanning and imaging capabilities heavily rely on focus control. The traditional focus tuning method restricts the miniaturization of optical systems due to its complex structure and large volume. The recent rapid development of MEMS varifocal optical elements has provided sufficient opportunities for miniaturized optical systems. Here, we review the literature on MEMS varifocal optical elements over the past two decades. Based on light control mechanisms, MEMS varifocal optical elements are divided into three categories: reflective varifocal mirrors, varifocal microlenses, and phased varifocal mirrors. A novel indicator is introduced to evaluate and compare the performance of MEMS varifocal optical elements. A wide range of applications is also discussed. This review can serve as a reference for relevant researchers and engineers.

## 1. Introduction

Since the invention of the transistor in 1947 and Professor Feynman’s famous speech, “There is plenty of room at the bottom” in 1959, miniaturization has become a significant development trend in science and technology. Nowadays, microelectronic devices have permeated into all aspects of people’s lives. People are constantly working towards miniaturization and high integration of various systems, particularly optical systems. Focus control plays a critical role in light regulation, widely used to adjust focal points in imaging and scanning systems or to direct light beams toward specific targets [1,2]. In particular, fast and high-precise focus control is required for studying subcellular dynamics, detecting electrical signals from large numbers of neurons [3,4,5], and high-speed laser processing [6,7]. The traditional method for focus control uses electromechanical devices to change the relative position of the components of the optical systems. Optical systems utilizing mechanical focus control suffer from large volumes, complex mechanical structures, and slow speed [8]. These drawbacks restrict the miniaturization of optical systems and make integrating them with other microdevices into a single chip difficult. Varifocal optical elements are the key to solving these problems.

Varifocal optical elements are tunable optical elements that can change the position of the focal point. Various varifocal optical elements have been developed. Prominent examples such as liquid crystal lenses, electro–optic ceramic lenses, Alvarez lenses, tunable acoustic gradient index lenses (TAG lenses), spatial light modulators (SLMs), liquid lenses, and metalenses constitute the current main technical approaches [2]. Although the promotion of the miniaturization process shows the benefits of applying them, they are still required for higher speed of focus control and cheaper cost of manufacturing.

The vigorous development of MEMS technology provides a new technical direction for varifocal optical elements, namely MEMS varifocal optical elements. MEMS varifocal optical elements are a combination of varifocal elements and MEMS technology, fabricated through a microfabrication process. Taking advantage of MEMS and microfabrication—miniaturization, microelectronic integration, and high-precision mass fabrication—these varifocal optical elements exhibit features such as micro or sub-micro scale dimensions, lightweight design, cost-effectiveness, and high-density integration with microelectronics [9,10]. Compared with similar technologies, MEMS varifocal optical elements enable the miniaturization of optical systems and simultaneously provide a wide varifocal range reaching up to 2000 D and a fast varifocal speed above several hundred kilohertz. These advantages provide them with broad application prospects. Lots of pioneering work in the application of MEMS varifocal optical elements has been demonstrated, ranging from optical zoom [11,12,13], high-speed 3-D scanning or display [14,15], biomedical stereoscopic imaging [16,17,18,19], minimally invasive therapy [20], optical sensors [21,22], and various laser applications [23,24,25].

In this review, we examine the recent progress of MEMS varifocal optical elements. Nearly a hundred designs based on various principles are summarized in four tables. These designs are categorized according to how they control light beams. In Section 2, we provide a detailed introduction to three main categories of MEMS varifocal optical elements along with a brief overview of the research progress. In addition, a new evaluation method for a fair comparison is described in detail. A description of applications that have already been realized follows in Section 3. Section 4 shows the comparison results using the proposed evaluation method. Finally, we conclude with a discussion and outlook for MEMS varifocal optical elements in Section 5.

## 2. MEMS Varifocal Optical Elements

**Figure 1:**
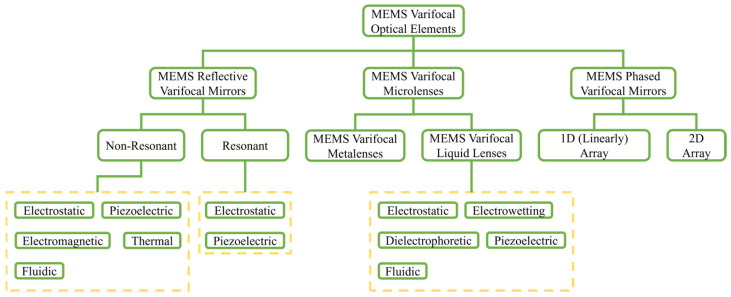
Descriptive diagram illustrating the classification situation. MEMS varifocal optical elements are categorized into three types: MEMS reflective varifocal mirrors, MEMS varifocal microlenses, and MEMS phased varifocal mirrors. Each type is further divided according to its unique characteristics.

According to the basic principles of optics, MEMS varifocal optical elements are classified into three major categories, including MEMS reflective varifocal mirrors, MEMS varifocal microlenses, and MEMS phased varifocal mirrors. Each category is further subdivided into subcategories. The general classification is described in Figure 1.

The research history of MEMS varifocal optical elements can be traced back to the 1980s. An electrostatic servo system was reported by Fujita et al. and experimentally used to drive a varifocal mirror in 1988 [26]. Subsequently, a non-uniform surface silicon diaphragm dynamic focusing mirror fabricated with microfabrication was proposed. It could reduce aberrations and improve the modulation transfer function by nearly twice [27]. Both designs used electrostatic actuators to deform the mirror surface. Burns et al. [28] first investigated a varifocal mirror that deformed the mirror surface through Joule heat. The mirror was composed of concentric rings of polycrystalline silicon and gold. Recognizing the benefits of Burn’s concentric ring structure, Himmer et al. introduced a silicon nitride variable focus mirror driven by electrostatic force [29]. Since then, reflective MEMS micromirrors specially designed for focal control have received increasing attention and have been applied in various optical devices, especially those that require rapid axial focusing. Another source of MEMS reflective varifocal mirrors is MEMS deformable mirrors (DMs). MEMS deformable mirrors are surface-deformable micromirrors, engineered to correct various optical aberrations for enhanced imaging quality [30]. MEMS reflective varifocal mirrors can be viewed as deformable mirrors providing defocus aberration compensation. Hence, utilizing MEMS deformable mirrors can realize focus control. Lenses, as one of the ancient optical elements, play an essential role in optics. Almost all optical systems cannot allow specific functions without using lenses to build light paths. Hence, developing and exploiting varifocal microlenses for focus control is a necessary and efficient technical direction for miniaturized optical systems. Many methods have been proposed to realize microlenses, such as Alvarez lenses, liquid lenses, and Metalenses. Considering the millimeter or sub-millimeter scale of microlenses, it is natural to combine them with MEMS technology [31]. Therefore, there has been great interest in conducting in-depth research on MEMS varifocal microlenses. Inspired by optical phased arrays, a novel method that modulates the phase of the light beam to form a spherical light wave has been proposed. Relevant research is still in its preliminary stages, but MEMS phased varifocal mirrors provide a flexible choice for focus control.

After years of development, MEMS varifocal optical elements have gradually matured and have been successfully implemented in diverse optical applications. Optical zoom systems utilizing MEMS varifocal optical elements provide a viable option for micro-cameras, especially those used in smartphones [11,12,13]. MEMS varifocal optical elements can offer a balance between axial and lateral scanning in three-dimensional imaging of living tissue, such as OCT microscopes [32,33] and confocal microscopes [17,18,19], and can improve imaging resolution. Meanwhile, MEMS varifocal optical elements capable of achieving three-dimensional scanning simultaneously provide another resolution for the mismatch between axial scanning speed and the increasingly rapid lateral scanning speed [34,35,36,37,38,39,40]. They can also be integrated into Head-Mounted Displays (HMDs) to generate stereoscopic images, enhancing user comfort with their lightweight design, as well as accommodating Vergence Accommodation Conflict (VAC) [15]. Furthermore, MEMS varifocal optical elements can be used to steer light beams for specific purposes such as optical communication [41], laser processing, or measurement [21,22,24]. Lastly, MEMS reflective varifocal mirrors and phased varifocal mirrors can be viewed as MEMS DMs, providing sufficient defocus aberration compensation for adaptive optical systems [42,43,44].

### 2.1. Physics Principle, Micro-Actuation Mechanism, and Evaluation Metrics

Prior to systematically examining the three principal categories of MEMS varifocal optical elements, this section provides the necessary fundamentals, encompassing a detailed analysis of the basic physics principles, an overview of micro-actuation mechanisms, related microfabrication techniques, and an innovative performance evaluation methodology.

#### 2.1.1. Physics Principle

The fundamental physical principles obeyed by MEMS varifocal optical elements are merely reflection, refraction, and diffraction. We take the circular element as an example to describe the physical principles of these three types, as most MEMS varifocal optical elements are circular in shape.

##### MEMS Reflective Varifocal Mirrors

**Figure 2:**
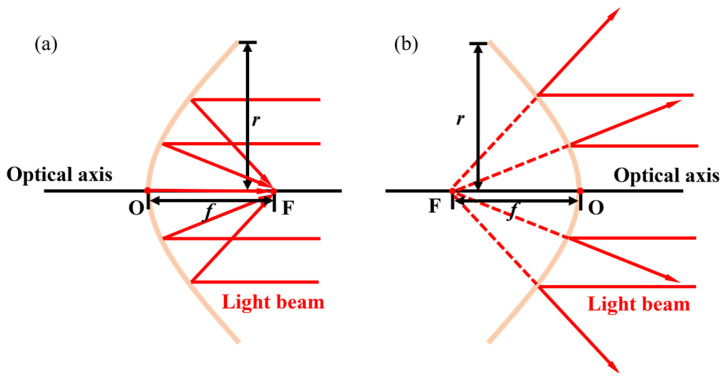
The physics principle of MEMS reflective varifocal mirrors: (**a**) the condition of light convergence; (**b**) the condition of light divergence.

As the name suggests, MEMS reflective varifocal mirrors are based on reflection. When operating a MEMS reflective varifocal mirror, the mirror surface deforms into a parabolic surface by converting electromagnetic or other energy into mechanical energy. Incident light reflects off the mirror surface at different angles, realizing the convergence of the reflected light. Figure 2 illustrates two conditions of an actuated MEMS reflective varifocal mirror. As shown in Figure 2a, the light beam that reflects off the mirror surface converges at a single point called the real focal point. In Figure 2b, the light beam diverges towards the vicinity after undergoing reflection. The reverse extension line of the light beam converges at a point referred to as the virtual focal point. In this review, we treat both as the focal point and do not make any distinctions between them. The focal point and the mirror vertex are marked with F and O separately. The distance between the mirror vertex O and the focal point F is the focal length represented by *f*. The focal length may be positive or negative. If the direction of a ray drawn from point O to point F is from left to right, we consider the focal length to be positive. On the contrary, the value is negative. It should be noted that the sign of focal length merely reflects the nature and position of the focal point. The formulas related to focal length in the following part are all calculated by substituting the numerical values of focal length.

MEMS reflective varifocal mirrors are more suitable for standard MEMS microfabrication processes. Utilizing reflective mirrors can fold the light path, so it provides choices to reduce the volume of the optical systems and flexibly direct light. In addition, reflection does not cause chromatic aberration, which enhances the convergence of light. There are also limitations for MEMS reflective varifocal mirrors. Mechanical structures may impact optical performance—for example, the pull-in effect in plate capacitor structures. The inherent characteristics of the utilized materials may also cause difficulties in using MEMS reflective varifocal mirrors, such as the hysteresis effect of piezoelectric materials, the stress characteristics of the materials, the reflectance of mirror materials, etc. Finally, some varifocal mirrors, such as electrostatic and piezoelectric mirrors, exhibit fundamental limitations in high-power laser systems due to their inherent intolerance to thermal effects.

##### MEMS Varifocal Microlenses

**Figure 3:**
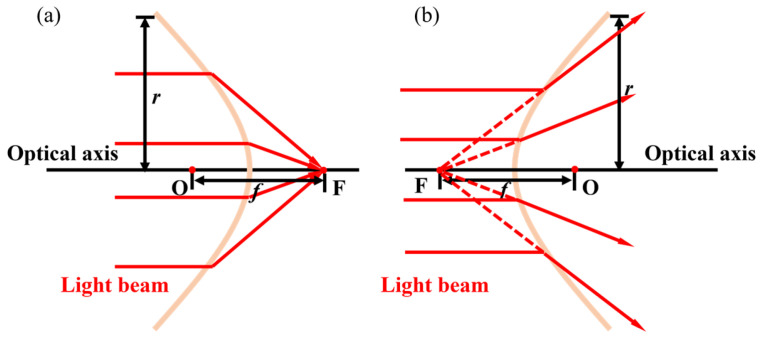
The physics principle of MEMS varifocal microlenses: (**a**) the condition of light convergence; (**b**) the condition of light divergence.

When light passes through MEMS varifocal microlenses, due to refraction, the light deviates from its original direction of propagation. Figure 3 depicts the two conditions of this deviation. Figure 3a shows that the parallel light beam refracts on the surface of the lens and converges at focal point F. While in Figure 3b, the reverse extension line of the light beam converges at focal point F. In this case, O represents the optical center of the lens. The distance between O and F is the focal length. Whether it is positive or negative still depends on the direction of the ray drawn from O to F.

MEMS varifocal microlenses are very important varifocal optical elements, as almost all optical systems employ lenses. There are many advantages to using them. Firstly, compared to MEMS reflective varifocal mirrors, the light energy loss of microlenses is relatively low. MEMS reflective varifocal mirrors rely on metal coatings to realize light reflection, which always suffers from absorption loss. MEMS varifocal microlenses adopt high-transparency materials such as glass and polymers, reducing optical energy loss. Secondly, some microlenses do not have any movable mechanical structures, which enhances their reliability. Especially when facing collision, they exhibit strong impact resistance capability. There are also some drawbacks. Many microlenses use liquid as filler, which poses challenges to microfabrication. Moreover, the varifocal speed is often slower. This makes MEMS varifocal microlenses applicable to optical systems that do not require rapid focus scanning, such as smartphone cameras. They cannot meet the demand for rapid focus scanning. In addition, microlenses often require compensation for aberration.

##### MEMS Phased Varifocal Mirrors

Both MEMS reflective varifocal mirrors and MEMS varifocal microlenses fundamentally adhere to the basic principles of geometric optics. Different from the two types, MEMS phased varifocal mirrors utilize diffraction. Broadly speaking, the diffraction of light can be understood as the alteration of the light’s wavefront caused by the restriction of its free propagation due to the presence of obstacles. Obstacles, commonly referred to as diffraction screens, can modulate the amplitude or phase of light. Consequently, the desired wavefront can be generated through the design of specific diffraction elements.

**Figure 4:**
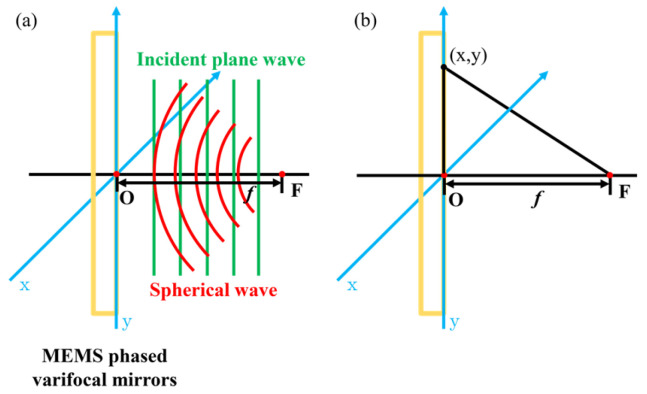
The physics principle of MEMS phased varifocal mirrors. (**a**) shows that when a plane optical wave passes through a MEMS phased varifocal mirror, the plane wave is altered into a spherical optical wave due to phase compensation. (**b**) describes the optical path length (OPL) of each element.

From the perspective of wavefront transformation, focusing fundamentally involves converting an incident plane wave into a spherical wave, as shown in Figure 4a. When an incident wave plane passes through the specific MEMS phased varifocal mirror, the MEMS phased varifocal mirror provides phase compensation for the optical wave. MEMS phased varifocal mirrors consist of many reflective elements. A Cartesian coordinate system is established on the initial surface of the undriven mirror. The position of each element can be determined by its coordinates. We assume that the element at position $\left(x,y\right)$ introduces a phase shift φ corresponding to the specified focal length *f*. The additional optical path difference $\left(OPD\right)$ induced by the $\left(x,y\right)$ element relative to the central element at the focal point can be expressed by Equation (1):

(1) $$OPD=[\sqrt{(x^{2}+y^{2})+f^{2}}-f]+\varphi /k$$

Here, k denotes the angular wavenumber given by Equation (2).

(2) $$k=\frac{2\pi }{\lambda }$$

λ is the wavelength of the incident optical wave. The resultant additional phase difference $\left(PD\right)$ is therefore given by:

(3) PD=kOPD

Analogous to Fresnel zone plates, the secondary wavelets generated by every element undergo constructive interference at the designated focal point. Therefore, the additional phase difference must satisfy the conditions specified in Equation (4).

(4) PD=2mπ(m=0,1,2,⋯)

To simplify the implementation of phase modulation, we set m=0. By substituting Equations (1) and (2) into Equation (3), we obtain φ as:

(5) $$\varphi =\frac{2\pi [f-\sqrt{(x^{2}+y^{2})+f^{2}}]}{\lambda }$$

Based on the Taylor expansion, we expand $\sqrt{(x^{2}+y^{2})+f^{2}}$ into Equation (6).

(6) $$\sqrt{(x^{2}+y^{2})+f^{2}}=f+\frac{x^{2}+y^{2}}{2f}-\frac{{\left(x^{2}+y^{2}\right)}^{2}}{8f^{3}}+\cdots$$

Given that MEMS phased varifocal mirrors are typically smaller than the designated focal length, the paraxial approximation is applied. Under the paraxial approximation, higher-order terms beyond the second in the expansion can be neglected. Thus, Equation (5) can be approximated as:

(7) $$\varphi =-\frac{\pi (x^{2}+y^{2})}{\lambda f}$$

We can see that Equation (7) describes the phase transfer function characteristic of a thin lens in the paraxial regime. So, introducing the phase shift decided by Equation (7) can ensure focus.

It should be noted that this phase modulation principle is not only applicable to MEMS phased varifocal mirrors, but also explains the varifocal mechanism of MEMS varifocal metalenses, which are classified as MEMS varifocal microlenses.

MEMS phased varifocal mirrors offer a more flexible way to achieve focus adjustment. The combination of reflective micromirror arrays and phase modulation provides considerable benefits. Since focus tuning utilizes phase modulation, the varifocal process can move beyond mechanical constraints, particularly support components. This advancement opens new possibilities in design and functionality. Due to the small pixel size, the advantages of MEMS can be fully utilized to achieve faster, deeper varifocal control and lower energy consumption. In addition, reasonable modulation can realize simultaneous targeting of multiple depths. However, since this phase modulation is based on diffraction, improper micromirror dimensions can result in sidelobes. This is similar to Optical Phased Arrays (OPAs). Additionally, the heat generated from densely packed mirror elements poses significant challenges for thermal dissipation design.

#### 2.1.2. Micro-Actuation Mechanism

Physical analysis demonstrates that both MEMS reflective varifocal mirrors and varifocal microlenses require actuation forces to generate controlled mirror deformations or lens reconfigurations. MEMS phased varifocal mirrors employ piston-type actuators to achieve precise phase compensation through controlled vertical displacement. MEMS actuators can deliver adequate actuation force to power micro-scale devices. Thus, specific geometric transformation or motion can be realized through the combination of the structure of MEMS varifocal optical elements and a targeted actuation mechanism.

The primary MEMS actuation mechanisms comprise electrostatic, piezoelectric, thermal, and magnetic actuators. Electrostatic actuators employ capacitive actuation principles to convert an input voltage into significant output motion. In general, electrostatic actuation has four advantages that can explain why it is commonly and widely used. Firstly, electrostatic actuators exhibit rapid response characteristics to applied voltages [45]. Secondly, it possesses the ability to generate significant force without requiring a high steady-state current [46]. This minimizes thermal output, effectively mitigating heat-induced damage risks. Meanwhile, it shows that the actuation mechanism has enhanced energy conversion efficiency, enabling low-power operation. Thirdly, capacitive architectures demonstrate inherent fabrication simplicity, offering exceptional compatibility with standard semiconductor processing techniques. However, there is a major drawback limiting the performance of electrostatic actuation. The electrostatic force typically exhibits a quadratic relationship with displacement, whereas the mechanical restoring force demonstrates a linear dependence on stroke. Actuator failure occurs when displacement exceeds one-third of the electrode gap, as mechanical restoring forces become insufficient to counterbalance electrostatic attraction in the capacitor system. This is referred to as the pull-in effect, which shows that the range of motion is restricted to one-third of the electrode gap. Electrostatic actuation produces unidirectional downward force without inherent upward driving capability. Additionally, multi-electrode structures can bring about coupling problems.

Piezoelectric actuators are based on piezoelectric materials. Electrical excitation of piezoelectric materials generates controllable mechanical deformation for actuation purposes. Piezoelectric actuation possesses plenty of advantages, making them more and more attractive [47]. Piezoelectric materials can generate significant force by contracting or expanding by a small amount. This characteristic enables a large stroke with a low applied power. Furthermore, they exhibit negligible thermal dissipation, resulting in significantly reduced power consumption. It also should be noted that high and stable resonant frequencies make fast and steady dynamic resonant operation possible. Despite their significant advantages, piezoelectric materials’ inherent hysteresis behavior fundamentally limits precision actuation performance through nonlinear displacement–voltage characteristics. The hysteresis characteristic in piezoelectric materials manifests as a nonlinear, path-dependent delay between applied stimuli (electric fields or mechanical stresses) and material responses (polarization or strain). This phenomenon creates characteristic hysteresis loops (ferroelectric P-E loops or butterfly-shaped S-E curves), where loading or unloading paths exhibit distinct trajectories rather than superposition. This hysteresis leads to inconsistent displacement outputs under identical driving voltages, critically compromising positioning accuracy in applications requiring sub-micrometer resolution. In addition, the performance ceiling of piezoelectric actuators in part stems from inherent process-induced material degradation during thin-film deposition and patterning in MEMS fabrication processes.

Thermal actuators are typically bilayer structures composed of two materials with different coefficients of thermal expansion. Electrical current through the actuator produces Joule heat, inducing bilayer structure bending via differential thermal expansion.

Thermal actuation is well known for its potential to achieve a large stroke. Like piezoelectric actuators, thermal actuators can generate considerable force through relatively small expansions or contractions. In addition, their geometric simplicity enables seamless integration with microfabrication processes. However, thermal actuation is limited by inherent drawbacks. First of all, it is difficult to obtain the relationship between the actuation force and the current flowing through. This is due to the complex thermodynamic equilibrium mechanism and the structure of MEMS thermal actuators. Secondly, many MEMS devices are not robust enough to withstand excessive heat, causing thermal damage. Furthermore, as the size decreases, it is easier to dissipate the heat. Consequently, thermal actuation always requires high power to operate. Finally, while miniaturization accelerates thermal response, thermal actuation remains comparatively slower than alternative actuation mechanisms.

Electromagnetic actuators typically consist of a coil and magnetic component. If current flows the coil, a Lorentz force is generated between the magnetic component and the coil. Electromagnetic actuation can exert a large force. Additionally, electromagnetic actuation requires fewer electrical connections than alternative actuation mechanisms. Current electromagnetic actuation systems face inherent miniaturization challenges due to their structural complexity—for example, the structure of the solenoid coil—creating particular obstacles in microfabrication processes. Meanwhile, the deposition and etching of magnetic materials also pose challenges to microfabrication processes.

Beyond these four major actuation approaches, fluidic actuation, utilizing controlled pressure differentials, provides an alternative actuation mechanism. Fluidic actuators typically consist of a well-sealed fluid chamber filled with a specific fluid. Injecting or withdrawing fluid from the chamber via micropumps or microinjectors creates pressure that serves as th actuation source. Using a fluidic actuator can obtain a large stroke. In addition, the liquid used in the actuator can act as a coolant, reducing thermal damage to the device. Hence, this makes fluidic actuation suitable for high-power laser applications. Furthermore, fluidic actuation is a natural and convenient actuation mechanism for some MEMS varifocal microlenses whose structure consists of liquid. However, slow response speeds severely constrain the practical implementation of fluidic actuation. Additionally, the presence of fluids creates substantial integration barriers between fluidic actuators and microelectronics. Meanwhile, introducing fluid also poses challenges for fabrication.

Apart from these actuation methods, certain MEMS varifocal optical elements require actuator structures specifically designed according to their unique actuation principles. For example, MEMS varifocal liquid lenses based on the electrowetting effect always need specific actuators capable of changing surface tension.

#### 2.1.3. Evaluation Metrics

This section introduces a novel performance indicator, *I.* We assume that all MEMS varifocal optical elements are ideal elements and will not cause any loss of light. To clarify the derivation of *I*, we also take the circular optical element as an example. Before presenting our proposed evaluation methodology in detail, we shall first review the conventional metrics employed for the performance assessment of MEMS varifocal optical elements. The focal length *f* can directly characterize the focus ability. Thus, the varifocal capacity can be quantitatively characterized by the focal length variation (Δf), which is defined as the difference between the maximum focal length and the minimum focal length, as shown in Equation (8).

(8) $$\Delta f=f_{\max}-f_{\min}$$

However, the two parameters are both unique for each MEMS reflective varifocal mirror because every mirror has its own specific focal length and differential focal length. Furthermore, if the focal length is infinite, the focal length variation cannot be quantified.

Optical power (OP), which is also referred to as dioptric power, is a better measure of an optical element’s ability to converge or diverge light. It is the reciprocal of the focal length, as shown in Equation (9).

(9) OP=1/f

Optical power offers a more reasonable and quantifiable method for performance characterization. From Equation (1), it can be found that optical power reflects how strongly the optical element can bend the light beam, thus indicating focusing ability. Like focal length, optical power can have a positive or negative value. Positive optical power represents the convergence of the light rays, while negative optical power represents the divergence.

Within the framework of optical power, we can use optical power variation to characterize the varifocal ability. Optical power variation (ΔOP) is defined as the difference between the maximum optical power and the minimum optical power, as shown in Equation (10).

(10) $$\Delta OP=OP_{\max}-OP_{\min}$$

While focal length variation and optical power variation effectively quantify variable focusing capability, they fundamentally characterize the magnitude of focal length adjustment. The two metrics neglect the fact that due to the effects of light diffraction, the actual focal point is not a perfect point but a light spot with both width and depth. On one hand, the variation-based metrics cannot adequately assess optical focusing quality. On the other hand, since light spots have depth, two light spots cannot be distinguished by the detector within the range determined by the Rayleigh criterion. Strictly speaking, this demonstrates that only discrete focal planes can be achieved rather than the ideal continuous focal length adjustment.

In order to be able to simultaneously and precisely measure the focusing effect and the varifocal effect, we propose a new indicator for MEMS varifocal optical elements. It also can be used to comprehensively evaluate and compare the performance of MEMS varifocal optical elements. Due to the influence of light diffraction, a set of concentric rings with alternating brightness and darkness will be generated at the focal point. A total of 84% of the energy is concentrated in the central circle, which is also known as the Airy spot. The Rayleigh criterion provides the radius and axial depth of the Airy spot, as shown in Equations (11) and (12) [48].

(11) $$R=\frac{1.22\lambda }{n\sin\alpha }=\frac{1.22\lambda }{NA}$$

(12) $$z=\frac{2\lambda }{{\left(n\sin\alpha \right)}^{2}}=\frac{2\lambda }{NA^{2}}$$

R,z,λ,n and α represent the radius of the Airy spot, the depth of the Airy spot, the wavelength of the incident light, the refractive index of the medium that surrounds the mirror or fills the lens, and aperture angle, respectively. nsinα represents the numerical aperture, denoted by NA. According to the geometric relationship, the sine value of the aperture angle can be expressed as:

(13) $$\sin\alpha =\frac{r}{f}$$

*r* represents the radius of the element. Thus, Equations (11) and (12) are rewritten as:

(14) $$R=\frac{1.22\lambda }{NA}=1.22\lambda \frac{f}{nr}$$

(15) $$z==\frac{2\lambda }{NA^{2}}=2\lambda {\left(\frac{f}{nr}\right)}^{2}$$

As mentioned above, not all depth information can be obtained within the range of focal point variation. The number of resolvable focal planes, $N_{\mathrm{planes}}$ [49], is introduced to characterize the varifocal effect. Equation (16) expresses $N_{\mathrm{planes}}$:

(16) $$N_{\mathrm{planes}}=\int_{f_{\min}}^{f_{\max}}\frac{df}{z}$$

Substituting Equation (15) into Equation (16), we obtain:

(17) $$N_{\mathrm{planes}}==\int_{f_{\min}}^{f_{\max}}\frac{n^{2}{\left(r/f\right)}^{2}}{2\lambda }df=\frac{n^{2}r^{2}}{2\lambda }\left(\frac{1}{f_{\min}}-\frac{1}{f_{\max}}\right)=\frac{n^{2}r^{2}}{2\lambda }\left(OP_{\max}-OP_{\min}\right)=\frac{n^{2}r^{2}}{2\lambda }\left(\Delta OP\right)$$

$N_{\mathrm{planes}}$ indicates the discrete varifocal effect, offering a precise description of varifocal ability. However, it cannot reflect the focusing effect R, the radius of the Airy spot, represents the quality of the light spot at the focal point. Similar to the calculation process of $N_{\mathrm{planes}}$, we define γ as the reciprocal of R to characterize the focusing effect. Equation (18) expresses γ:

(18) $$\gamma =1/R=\frac{nr}{1.22\lambda f}$$

This aligns with our intuition—the larger γ is, the higher the focusing quality it represents. To assess both varifocal capability and focusing quality simultaneously, we propose an evaluation indicator I by introducing γ into the calculation of $N_{\mathrm{planes}}$, as shown in Equation (19)

(19) $$I=\int_{f_{\min}}^{f_{\max}}\gamma \cdot \frac{df}{z}=\int_{f_{\min}}^{f_{\max}}\frac{nr}{1.22\lambda f}\frac{{\left(nr/f\right)}^{2}}{2\lambda }df$$

By retaining only the parameters relevant to the element itself, we obtain the following Equation (20). Thus, in this review, we utilize $r^{3}\Delta OP^{2}$ to represent I.

(20) $$I\propto \int_{f_{\min}}^{f_{\max}}{\left(\frac{r}{f}\right)}^{3}df=r^{3}\left(\frac{1}{f_{\min}^{2}}-\frac{1}{f_{\max}^{2}}\right)=r^{3}\left(OP_{\max}^{2}-OP_{\min}^{2}\right)=r^{3}\Delta OP^{2}$$

We noticed that using I can evaluate the performance of the MEMS varifocal optical element, which is conveniently circular. However, when the element shape is rectangular or elliptical, we modify the expression of I:

(21) $$I=r_{\mathrm{effective}}^{3}\Delta OP^{2}$$

Most optical elements are circularly symmetrical. The optical theory is also based on circular optical elements. Hence, the optically functional region is always circular. $r_{\mathrm{effective}}$ represents half the dimensions of the optically functional region. For circular optical elements, $r_{\mathrm{effective}}$ is always the radius of the element. However, for non-circular elements, the situation becomes somewhat complicated. To ensure compatibility with the established optical theory, an equivalent circular domain is defined to just cover the practical optical-function region. $r_{\mathrm{effective}}$ represents the radius of this equivalent circular domain. To determine $r_{\mathrm{effective}}$, we envision a scenario where a non-circular MEMS varifocal optical element is employed in microscopes. This element has a short side and a long side. If we choose to have the equivalent circular domain completely cover the long side, then light will propagate from the vicinity of the short side to other instrument components. This will generate stray light, seriously interfering with the imaging quality of the microscope. Thus, in order to ensure the high utilization of light energy, as well as avoid the occurrence of stray light, the optically functional region should cover the short side. Hence, $r_{\mathrm{effective}}$ represents half the length of the short axis or short side.

Considering that some MEMS varifocal optical elements are only used at the resonant frequency, we combine the resonant frequency with I [49].

(22) $$I_{resonance}=r^{3}\Delta OP^{2}f_{\mathrm{resonance}}$$

### 2.2. MEMS Reflective Varifocal Mirrors

In this section, MEMS reflective varifocal mirrors are categorized according to actuation approaches. The actuation approach is defined according to the type of driving signal applied. In general, micromirrors use two approaches: digital and analog [50]. MEMS reflective varifocal mirrors use the analog approach due to the requirement for continuous focus control. There is a special case that should be noted. In this case, the mirror is actuated by a harmonic signal whose frequency is set to its natural frequency. This induces resonance phenomena, where compared to actuation by electrical signals at other frequencies, the motion of the MEMS reflective varifocal mirror is substantially amplified at this specific frequency. Based on this principle, numerous MEMS reflective varifocal mirrors specifically driven by resonant signals have been developed. Therefore, we classify MEMS reflective varifocal mirrors into two types: non-resonant and resonant.

#### 2.2.1. Non-Resonant

Most MEMS reflective varifocal mirrors are non-resonant. Non-resonant MEMS reflective varifocal mirrors are divided into five types: (1) electrostatic, (2) piezoelectric, (3) thermal, (4) electromagnetic, and (5) fluidic. This classification is reasonable, as mirrors utilizing the same actuation mechanism possess similar structures. Table 1 provides a summary of the five types developed from 2000 to 2024.

##### Electrostatic Actuation

**Figure 5:**
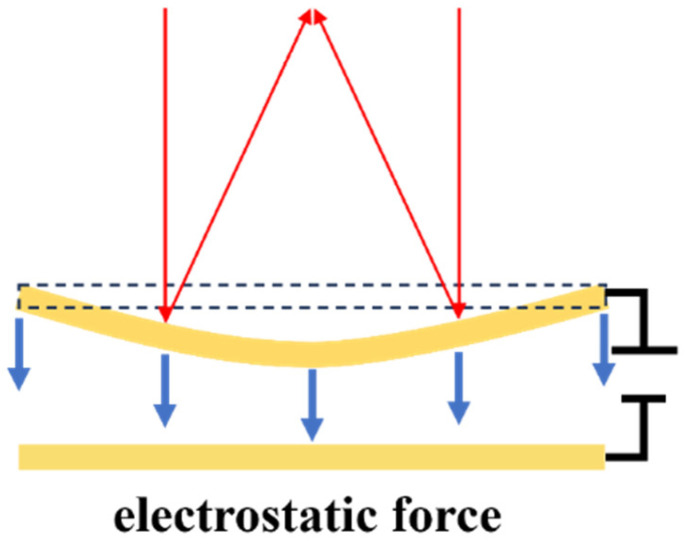
Schematic diagram of electrostatic actuation. Electrostatic attraction generated between two electrodes attracts the mirror plate downwards, resulting in paraboloidal deformation.

**Figure 6:**
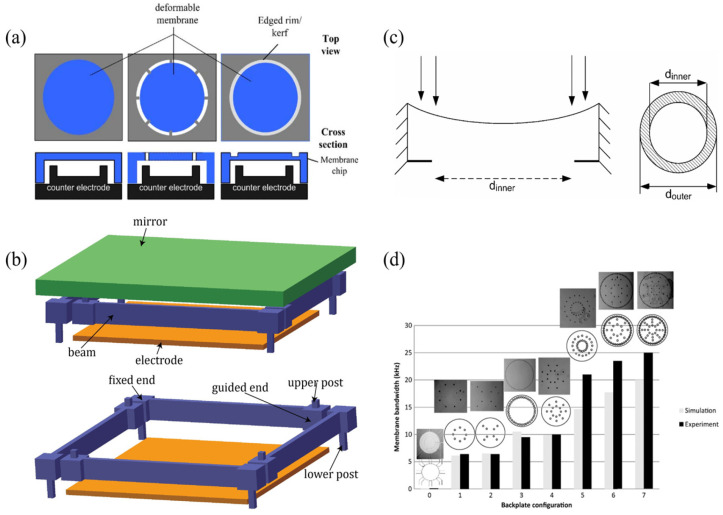
(**a**) Soft support as a compromise solution for a simply supported condition. Reprinted with permission from Ref. [52]. Copyright 2006 ELSEVIER. (**b**) Piston mirror with Quasi-Simply Supported piecewise linear flexure. Reprinted with permission from Ref. [68]. Copyright 2022 IEEE. (**c**) Ring-shaped electrode to address pull-in limitations. Reprinted with permission from Ref. [35]. Copyright 2006 ELSEVIER. (**d**) Vertical air channels distributed beneath the membrane to expand bandwidth. Reprinted with permission from Ref. [61]. Copyright 2013 IEEE.

Similar to a parallel plate capacitor, the electrostatic structure consists of a mirror plate as a moving electrode and a stationary electrode fabricated below the mirror plate. As depicted in Figure 5, electrostatic attraction generated between two electrodes attracts the mirror plate to move downwards and causes paraboloidal deformation. Different voltages generate different parabolic surfaces, thus altering the position of the focal point. In the past twenty years, efforts have been mainly directed to design structures and enhance their functions. Figure 6 illustrates various methods developed to improve performance.

The surface profile is critical to the performance. A parabolic surface is the most desirable shape for only one focal point [68]. If the mirror surface is a non-paraboloid, the reflected light focuses at different points along the optical axis, causing spherical aberration [65]. By theoretical calculation and analysis, Himmer et al. [29] indicated that a simply supported membrane can achieve paraboloidal deflection. They designed thinned and segmented (an effective 10% duty width) mirror perimeter to approximate simply supported boundary and achieved spherical-aberration-free focus control from 36 to 360 mm. As a simply supported boundary is practically impossible, Mescheder et al. [52] proposed soft support as a good compromise, realized by a membrane suspended with thin beams or a thinned rim, as shown in Figure 6a. They also introduced a ring-shaped electrode for enhancement. In combination with the ring-shaped electrode, soft support allowed for a λ/10 shape error with an extended optical aperture above 5 mm. Ref. [67] presented a structure suspended with beams running tangentially to the membrane. The optimized structure relaxed intrinsic stress without deforming the membrane, resulting in wavefront errors between λ/5–λ/10 with a 5mm large aperture. Wang et al. [68] proposed a piston mirror with Quasi-Simply Supported piecewise linear flexure, as shown in Figure 6b. When an increasing voltage was applied, the mirror surface changed in two steps: downward movement and concave deformation. In the second step, the mirror was supported only at its four corners, therefore paraboloidal deformation could be obtained. Stefano and Luca [42] developed a push–pull membrane mirror featuring bidirectional electrostatic actuation via top-mounted transparent indium-tin-oxide (ITO) electrodes and bottom actuators. Experimental characterization revealed a defocus wavefront modulation capability of 2.6 μm peak-to-valley (PV) amplitude with 0.97 Zernike polynomial purity, confirming sub-λ/20 deviation from ideal parabolic profiles. This precision demonstrates robust focus control applicable to adaptive optics scenarios requiring dynamic aberration compensation, such as ocular wavefront correction.

Limited stroke is another serious factor affecting performance. The pull-in effect caused by the typical parallel-plate structure constrains the motion of electrostatic mirrors. The mentioned ring-shaped electrode [35] can also overcome this limitation. As presented in Figure 6c, because the downward shift of the mirror center was induced by the movement of the mirror edge rather than electrostatic attraction directly, the pull-in effect was essentially resolved. Ref. [54] described a method for forming a closed-loop feedback control system, using capacitive sensing and modulating the frequency of a coupled ring oscillator through a differential measurement technique. The proposed feedback scheme extended the range of motion to 75% of the air gap, beyond the limit of previous open electrostatic instability, which was one-third of the air gap.

Approaches to improve varifocal speed and enlarge bandwidth have also been studied. Moghimi et al. [8] created air channels to allow airflow into and out of the space under the membrane for relieving air reactive pressure. They reported a 3 mm varifocal mirror consisting of a metalized SU-8 membrane bonded to a silicon backplate where actuating electrodes were patterned. The experimental test demonstrated a 30 kHz first mechanical resonant frequency but an effective bandwidth of only 100 Hz. To improve the narrow bandwidth attributed to the damping of viscous airflow, a new design with vertical air channels distributed beneath the membrane was fabricated, as shown in Figure 6d. Transient response was increased by 250 times, and bandwidth was extended to 25 kHz.

##### Piezoelectric Actuation

**Figure 7:**
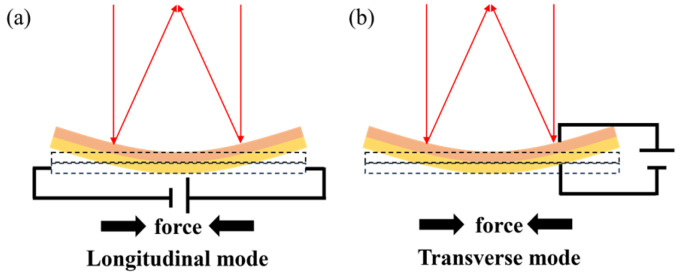
Schematic diagram of piezoelectric actuation: (**a**) longitudinal scheme; (**b**) transverse scheme. The contraction or expansion of the piezoelectric layer causes deformation of the mirror.

Piezoelectric types utilize the piezoelectric effect to deform mirror surfaces. When applying a voltage (or current) across the piezoelectric layer, it contracts or expands itself, and then a significant force can be exerted on the mirror layer. The generated force is uniform and parallel to the mirror layer so that it squeezes or stretches the mirror layer to deform the surface. Different forces induced by different voltages will deform the mirror surface into various parabolic surfaces. As shown in Figure 7, the current structure mainly has two schemes: the transverse mode and the longitudinal mode.

**Figure 8:**
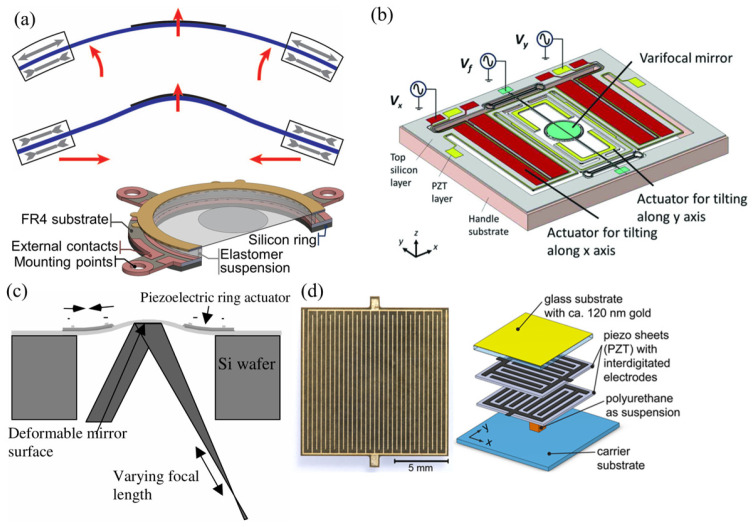
Selected examples of piezoelectric MEMS reflective varifocal mirrors: (**a**) The sandwich structure mirror consists of two piezoelectric layers and a mirror layer. Reprinted with permission from Ref. [71]. Copyright 2018 Optica Publishing Group. (**b**) Proposed piezoelectric mirror allowing lateral and axial scanning. Reprinted with permission from Ref. [40]. Copyright 2024 IEEE. (**c**) Iris-shaped piezoelectric mirror. Reprinted with permission from Ref. [69]. Copyright 2002 IEEE. (**d**) Varifocal mirror using two piezoelectric actuators, whose finger structures are oriented. Reprinted with permission from Ref. [70]. Copyright 2013 IEEE.

Transverse mode utilizes the transverse piezoelectric effect where the polarization direction is vertical to the deformation direction. The scheme is always attractive because it allows simple planar electrodes to drive the piezoelectric layer [69]. Walper et al. [71] proposed a thin glass membrane sandwiched by two piezoelectric rings, as shown in Figure 8a. The membrane exhibited convex deformation when the upper and lower rings contracted or expanded. When the two rings were in opposite states, the membrane bent into a concave shape. A fabricated 10 mm mirror could realize an optical power variation of more than 8 D and a high-quality image with a wavefront error between 0.5 and 1.2 μm. Inagaki et al. [72] described a mirror capable of both axis and lateral optical scanning. The device had a 4 mm mirror bonded with a PZT actuator for focus tuning. A pair of PZT actuators for mirror torsion was monolithically integrated into the outer frame of the device. This mirror can provide a maximum optical scanning angle of 26.2° at a 1.6 kHz resonant frequency, and a wide focus length range from −120 to +140 mm. As shown in Figure 8b, Sasaki et al. [40] also reported a micromirror operated with PZT thin films. The resonant frequencies for tilting along the x-axis (slow axis modes) and y-axis (fast axis modes) were 3.5 kHz and 11.6 kHz, respectively, with mirror torsion angles of 5° and 11.5°, respectively. In addition to scanning performance, it has a 115 mm focus tunable range and a few tens of microseconds response time. Sven et al. [43] developed a rapid focal shifter based on a unimorph deformable mirror. The device consisted of a piezoelectric material (PIC151) bonded to an ultra-polished glass substrate (N-BK10). Its surface was coated with a high-reflectivity dielectric film, while the bottom piezoelectric disk was patterned with one central electrode and eight concentric annular electrodes. Combined with a 250 mm focal-length f-θ lens, this focal shifter achieved a 68 mm longitudinal focal displacement. The operating bandwidth reached 2 kHz, with a 0.3 ms response time. This technology overcomes the bandwidth limitations of conventional mechanical focusing systems, providing a high-precision dynamic solution for high-speed laser 3D machining.

The longitudinal mode is based on the longitudinal piezoelectric effect, where the direction of an exerted electric field is parallel to the deformation direction of the piezoelectric layer. Considering that the longitudinal piezoelectric coefficient is usually twice that of the transverse piezoelectric coefficient, the longitudinal scheme can provide a larger stroke. Based on the scheme, Mescher et al. [69] reported a focusing micromirror. The device was an iris-shaped structure consisting of a poly-Si membrane and a ring-shaped piezoelectric actuator, as shown in Figure 8c. The iris-shaped mirror proved a varifocal range of several hundred microns and low MHz speed. The substantial tuning range and speed suggested that it could meet rapid focal length tuning requirements for scanning systems. Stürmer et al. [70] proposed a novel piezoelectric actuator that employed interdigitated electrodes on the piezoelectric layer to exert an electric field. They designed a prototype mirror that consisted of three layers: two stacked piezoelectric layers with perpendicularly oriented finger structures, and one mechanically passive mirrored glass layer, as shown in Figure 8d. The mirror surface allowed elliptic parabolic surface deformation as the voltage ratio of the upper to lower piezoelectric layer ranged from 0.58 to 1.86. The result suggested the potential for off-axis focus.

##### Thermal Actuation

**Figure 9:**
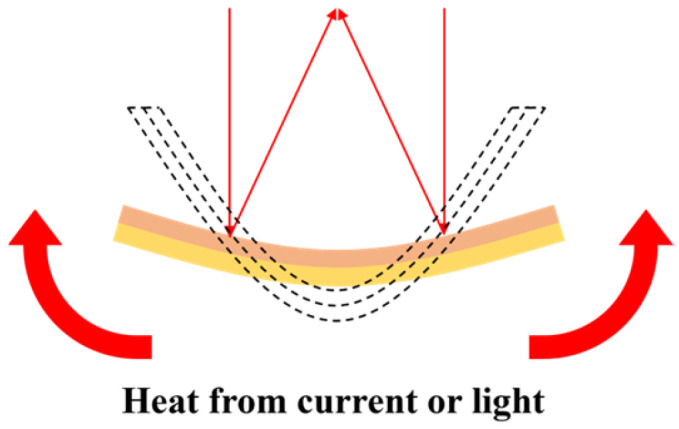
Schematic diagram of thermal actuation. The mirrow structure consists of two layers with distinct thermal expansion coefficients. When a temperature change occurs, a change in thermal stress leads to mirror deformation.

The core principle of thermal actuation is the use of heat to control stress. Thermal MEMS reflective varifocal mirrors are always composed of two layers of materials with significantly distinct thermal expansion coefficients. Due to the uniform distribution of initial residual stress, the mirror surface deforms into a parabolic surface before being driven. A temperature change in the mirror can change the shape of the mirror surface. The required heat for the temperature change is generated by applying a voltage (or current) to the thermal actuator or directing a high-power laser at the bottom of the mirror plate. When heat is transferred to the mirror, its surface will undergo new deformation due to the different expansions of the two layers, as depicted in Figure 9.

**Figure 10:**
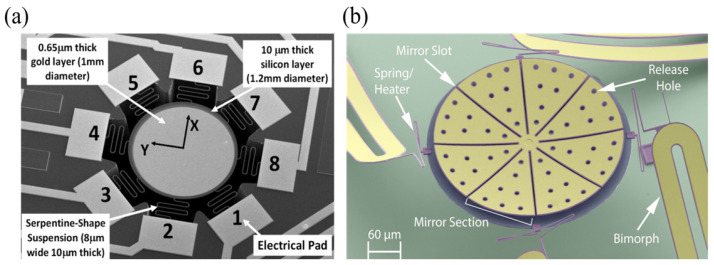
(**a**) Bimorph varifocal micromirror suspended by eight serpentine springs. Reprinted with permission from Ref. [74]. Copyright 2015 IEEE. (**b**) Thermal mirror connected to four thermal bimorphs via serpentine springs. Reprinted with permission from Ref. [41]. Copyright 2017 Optica Publishing Group.

Uttamchandani et al. [73] designed a gold–silicon bimorph varifocal micromirror suspended by eight serpentine springs, arranged around the edge of the mirror plate in an equally spaced radial pattern, as shown in Figure 10a. When a current flowed from one or more electrode pad(s) through corresponding serpentine spring(s) and mirror to another pad(s), Joule heat was generated. Alternatively, when a laser beam was directed to the rear surface of the mirror, the temperature could be changed by absorbing optical radiation. Considering that serpentine springs cannot withstand high temperatures and the mirror can only increase the temperature from ambient temperature, they actuated the mirror thermoelectrically with a Peltier element [74]. The thermoelectric properties of the Peltier element allowed the temperature to change from 10 to 100 °C without limitation from the ambient environment. Adopting this improvement approach also contributed to a 57% improvement in the achievable varifocal range. Morrison et al. [36] developed a mirror that combines lateral scanning capability, as shown in Figure 10b, realizing a varifocal range of −0.48 to 20.5 mm and a scanning angle of ±40°. Theoretical experiments demonstrated its capability for optical communication [41]. Marie et al. [43] proposed a thermally deformable mirror (TDM) composed of a high-reflectivity mirror substrate and resistive arrays bonded to its rear surface. It enabled thermal modulation of both substrate deformation and refractive index. The experimental results demonstrated the capability of TDM to correct defocus aberrations with ±30 m curvature radii (44 nm RMS) and tilt errors up to 50 μrad. Implementation of closed-loop algorithms with Zernike mode decomposition (first 20 orders) enhanced mode-matching efficiency from 97% to >99%, validating its capacity to dynamically optimize focal points through wavefront curvature modulation. This provides a non-mechanical focusing solution for gravitational wave detectors and related systems.

##### Electromagnetic Actuation

**Figure 11:**
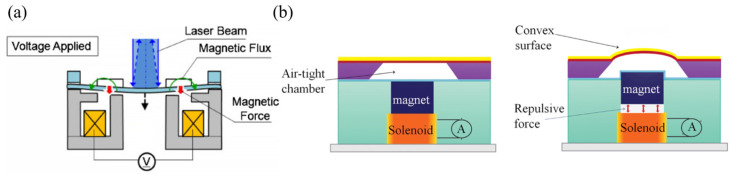
(**a**) Configuration of mirror using a non-contact electromagnetic actuator. Reprinted with permission Ref. [75]. Copyright 2011 IOP Publishing. (**b**) Schematic diagram of convex mirror and varifocal mechanism. Reprinted with permission from Ref. [76]. Copyright 2011 Optica Publishing Group.

The basic structure of electromagnetic MEMS reflective varifocal mirrors consists of a mirror plate and an electromagnetic actuator. The electromagnetic actuator can easily generate large forces. When the actuator is operated, the generated electromagnetic force directly changes the mirror surface or causes other mechanical forces through intermediaries to induce mirror deformation. Hashizume et al. [75] introduced a novel non-contact electromagnetic actuator for a deformable mirror, as shown in Figure 11a. It consisted of two components: a mirror element with a magnetic ring placed on it and an electromagnet placed underneath the mirror element. The mirror deforms into a paraboloidal shape without any physical contact when the magnetic field is generated between the magnetic ring and the electromagnet. Measurements showed a maximum deviation of only 30 nm from the ideal paraboloidal, demonstrating strong capability to compensate for spherical aberration. As depicted in Figure 11b, Hossain et al. [76,77,78] proposed an electromagnetic mirror used as a convex reflective mirror. The electromagnetic force generated between the magnet and solenoid pushed the magnet upward into the air-tight chamber. Due to the airtightness of the air chamber, the air inside the chamber could not leak outside. Eventually, air pressure was uniformly applied upward onto the mirror surface. Electromagnetic energy provided by the electromagnetic actuator was ultimately converted into mechanical energy of the mirror surface through the air in the chamber, thereby achieving parabolic deformation of the mirror surface. The system supports a 20 D optical power range.

##### Fluidic Actuation

**Figure 12:**
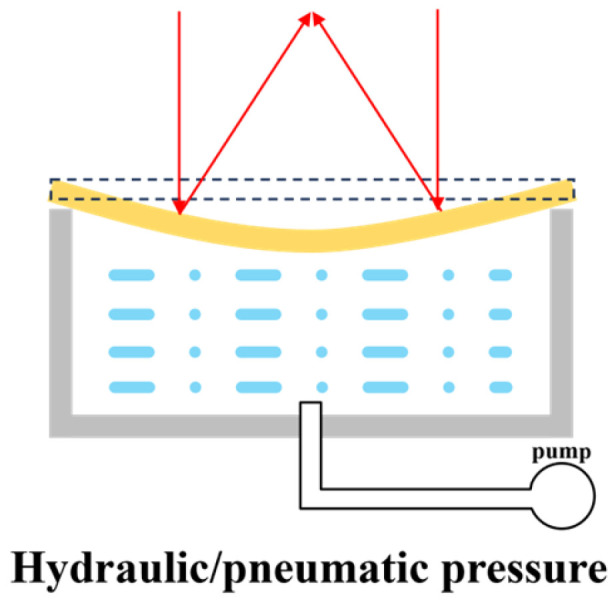
Schematic diagram of fluidic actuation. When micropump or microinjection pulls fluid in or out of the chamber, the pressure difference on the mirror plate results in deformation.

Figure 12 shows the principle of fluid MEMS reflective varifocal mirrors. A sealed fluidic chamber (often liquid) is always located under the mirror plate. Using microinjection or other devices to draw fluid in or out changes the volume of the fluid. It causes the pressure difference between the pressure exerted by the fluid and atmospheric pressure. Continuously changing the pressure difference induces parabolic deformation of the mirror surface. Geraldes et al. [20,79,80] manufactured a 16 mm^2^ varifocal mirror with a structure similar to that shown in Figure 10. The experimental results demonstrated a varifocal range of approximately 66 D and a 5% stabilization time of 398 ms. They also fabricated an off-axis reflective parabolic mirror and demonstrated its feasibility for an endoscope laser system.

#### 2.2.2. Resonant

Resonant MEMS reflective varifocal mirrors mainly utilize two actuation mechanisms: electrostatic and piezoelectric. Table 2 summarizes resonant MEMS reflective varifocal mirrors.

**Figure 13:**
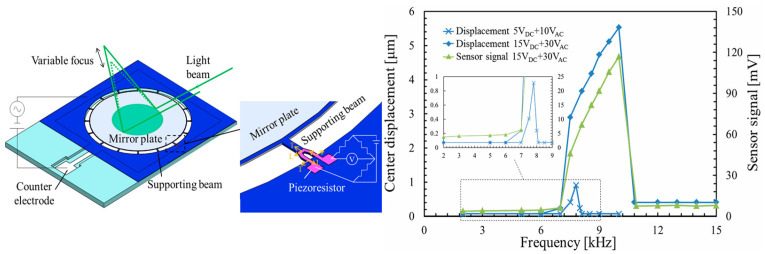
Schematic diagram of the resonant mirror. The frequency response of the mirror is also presented. Reprinted with permission from Ref. [81]. Copyright 2011 MDPI.

Sasaki et al. [63] first described a displacement-amplified dynamic varifocal mirror. The electrostatically actuated mirror was fabricated by bonding the deformable silicon membrane and electrode. In resonant mode, the dynamic stroke was amplified 50 times in a vacuum. Nakazawa et al. [81] reported a resonant MEMS varifocal mirror, as shown in Figure 13. The mirror consisted of a mirror plate suspended with sixteen U-shaped beams and a counter electrode deposited on a glass substrate. The unique design was an integrated piezoresistive focus sensor, fabricated by ion implantation on a beam of the mirror plate, which is used to monitor the focal length. The mirror center displacement at a frequency of 10 kHz was 92 times larger than the lower frequency from 2 to 6 kHz. Sasaki et al. [82] also developed a high-speed large-amplitude resonant varifocal mirror. They designed two electrostatic MEMS varifocal mirrors: circumference-supported (CS) and node-supported (NS) types. Under 1 Pa vacuum, vibration characteristics were measured via laser Doppler vibrometry with applied voltages of 30–150 V. The results demonstrated that the NS mirror achieved a maximum displacement amplitude of 1490 nm at 462.73 kHz, with a quality factor (Q) of 5324 (at 30 V), significantly higher than the CS mirror (Q = 2237). At 150 V, the NS mirror generated a focal length variation of ±28 mm. These validate that node support reduces energy loss and enhances resonant frequency and amplitude, enabling high-performance dynamic focus adjustment for laser machining and microscopy applications. Kocer et al. [83] proposed a MEMS continuous deformable mirror resonant based on electrostatic actuation (REA). The mirror features a 1.6 mm-diameter gold-coated silicon membrane anchored by eight support beams, with four concentric tiers of electrodes patterned beneath. Utilizing laser Doppler vibrometry, the first three resonant modes—defocus (16 kHz), primary spherical (81 kHz), and secondary spherical (187 kHz)—were measured, aligning closely with FEM simulation results. By adjusting the phase angle between the driving signal and laser pulses, the radius of curvature changed from 0.39 m to −7.2 m, achieving an 8 cm focal shift in an adaptive optics system. This design simplifies electrode control via resonant actuation, enabling high-speed focus modulation without complex algorithms.

**Figure 14:**
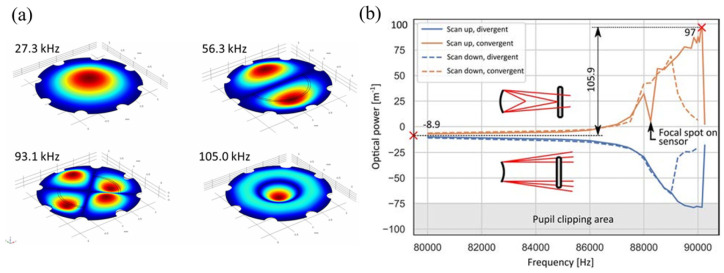
(**a**) Four resonant modes of the mirror demonstrated by FEM simulation. Reprinted with permission from Ref. [85]. Copyright 2018 IEEE. (**b**) Measured frequency response under AC 5 V with a DC 5 V offset. Reprinted with permission from Ref. [86]. Copyright 2024 IEEE.

Because piezoelectric materials typically achieve optimal mechanical performance when a resonant signal is applied, it is natural to utilize piezoelectric actuators for resonant mirrors. Ref. [85] showed a piezoelectric mirror whose frequency response demonstrated four resonant modes, as shown in Figure 14a. In the first and fourth resonant modes measured at 26.1 kHz and 107 kHz separately, the mirror surface had a spherical deformation, allowing fast control of the focus position. Pribošek et al. reported a design that features four equally spaced annular electrodes. The outer radii of each electrode were 25, 50, 75, and 100% of the mirror plate radius [86]. In resonant mode, the mirror could achieve a total optical power of ±100 D at 90 kHz as depicted in Figure 14b. They introduced a figure of merit (FOM), which is the product of mirror diameter, frequency, and optical power, to compare the performance of the state-of-the-art devices. The FOM of 24,780 verified that the performance of the proposed mirror far exceeded most others. Later, a new device excited with thin film piezoelectric actuators was reported in Ref. [87]. The critical feature was the combination of a binary Fresnel zone plate (BFZP) onto the flexible membrane. The mirror could introduce two additional focus points, resulting in four focusing points per harmonic cycle, thereby doubling the resonant frequency.

The benefits of operating in resonant mode are twofold. First, it can achieve a large stroke under a low voltage. When a harmonic driving signal whose frequency is close to the natural frequency of the mirror is applied, the amplitude of motion is significantly amplified. Second, the resonant frequency is always high enough to satisfy the demand for fast varifocal speed. In short, resonant MEMS reflective varifocal mirrors can achieve fast and wide varifocal with minimal power. The performance of resonant MEMS reflective varifocal mirrors is affected by energy loss due to viscous dumping, acoustic radiation, thermoelastic damping, support loss, and other factors [70]. It is critical to design proper structures to reduce energy loss. In addition, it is necessary to consider whether it matches other optical functions.

### 2.3. MEMS Varifocal Microlenses

MEMS varifocal microlenses are a combination of MEMS actuators and focus tunable microlenses. Many MEMS varifocal microlenses based on various principles have been proposed, such as Alvarez lenses [88], polymer lenses [89,90,91], Metalenses [92,93,94,95,96,97,98,99], and liquid lenses [21,100,101,102,103,104,105,106,107,108,109,110,111,112,113,114,115,116,117,118,119,120,121,122,123,124,125]. There are two common strategies for MEMS varifocal microlenses: MEMS varifocal metalenses and MEMS varifocal liquid lenses.

#### 2.3.1. MEMS Varifocal Metalenses

Metasurfaces are two-dimensional artificial planar materials composed of sub-wavelength microstructures arranged periodically. Due to the scale of microstructures being smaller than the wavelength of the incident light, modulation of amplitude, phase, and polarization can be realized. Therefore, it is possible to fabricate metasurface lenses (also called metalenses) for focus tuning. Modulation depends on either the material properties of microstructures or structural design [126]. Although changing the refractive index of microstructures is straightforward, large energy consumption is a fatal flaw for it. Hence, changing the structure (structural reconfiguration) is the mainstream. Introducing MEMS into structural reconfiguration metalenses provides opportunities to reduce the size and improve the varifocal speed. In the past ten years, many MEMS varifocal metalenses have been proposed. A summary is provided in Table 3.

**Figure 15:**
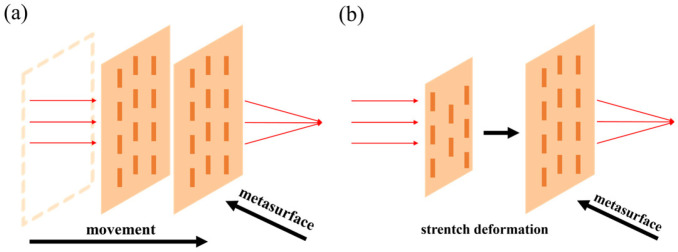
Schematic diagram of MEMS varifocal metalenses: (**a**) structural reconfiguration; (**b**) stretching metasurface.

There are two approaches for structural reconfiguration offered by relevant literature, as shown in Figure 15.

**Figure 16:**
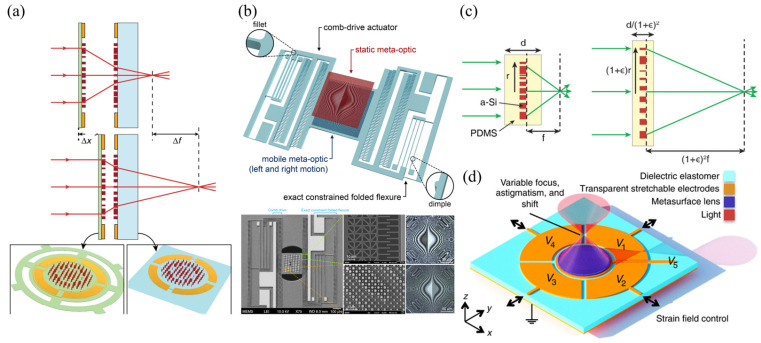
(**a**) MEMS varifocal metasurface doublet lens. Reprinted with permission from Ref. [93]. Copyright 2018 Springer Nature. (**b**) MEMS varifocal metasurface Alvarez lens. Reprinted with permission from Refs. [95,96]. Copyright 2020 and 2022 Springer Nature. (**c**) MEMS varifocal metalens based on DEA material. Reprinted with permission from Ref. [92]. Copyright 2016 Wiley. (**d**) MEMS varifocal metalens with specially designed electrodes. Reprinted with permission from Ref. [94]. Copyright 2018 AAAS.

The first approach uses two metasurfaces to form a doublet or Alvarez lens. MEMS actuators change the relative position (longitudinal or transverse movement) between two metasurfaces to adjust the focal point. Arbabi et al. [93] proposed a doublet lens with adjustable focal length, as illustrated in Figure 16a. The metasurfaces were fabricated on a glass substrate and SiN_x_ membrane, each with a periodic arrangement of α-Si nano posts acting as high-index dielectric scatterers. They used the two metasurfaces as a stationary lens and a moving lens. Attracting the moving lens towards the stationary lens causes a change of interval between the two lenses. Different intervals caused incident light to converge at different positions. The varifocal scheme proposed by Han et al. is similar to this. They demonstrated a MEMS varifocal metalens exploiting the Alvarez lens principle [95,96,97]. The device consisted of two phase-complementary metasurfaces made from an SOI wafer and a double-side polished (DSP) wafer, which served as a moving and stationary lens. As microstructures, SiNx nano posts were arranged on the substrate according to specific principles to form metasurfaces. The comb-drive actuator was also fabricated on the SOI wafer. After aligning and bonding the two metalenses, the MEMS Alvarez lens formed, as shown in Figure 16b. When a voltage was applied to the comb-drive actuator, the lateral relative movement of the two metalenses could be realized, permitting incident light to focus on other positions different from the initial position.

The other approach directly utilizes MEMS actuators to stretch the metasurface. Hence, the periodic arrangement of microstructures is rearranged. Kamali et al. [92] reported a Polydimethylsiloxane (PDMS) metasurface containing nano-columns composed of α-Si and Al_2_O_3_ arranged in a certain order inside. Stretching the metasurface changed the focal length, as shown in Figure 16c. When the metasurface stretched with a stretch ratio of 1 + ε, its focal distance changed by (1 + ε)^2^, theoretically demonstrating its ability to provide a wide varifocal range. As proof of concept, a 0.2 mm aperture lens was fabricated, and the experiment showed that the focal length varied from 600 to 1400 μm through radial strain. Based on the dielectric elastomer material, which can stretch when an electric field is applied, She et al. [94] designed a circular electrode consisting of a central circle and four peripheral fan-shaped rings. When a voltage was applied to the central electrode or the same voltages were applied to the four surrounding electrodes, the metasurface located on the electrode stretched as the electrode stretched. Figure 16d describes this process. The rearrangement of microstructures directed incident light to converge at different positions.

MEMS varifocal metalenses are novel and prospective methods for focus tuning. The combination of the planar structure of metasurface and MEMS technology will bring greater improvements to the performance of micro-optical systems. MEMS varifocal metalenses will make a substantial impact on the research progress of MEMS varifocal optical elements.

#### 2.3.2. MEMS Varifocal Liquid Lenses

**Figure 17:**
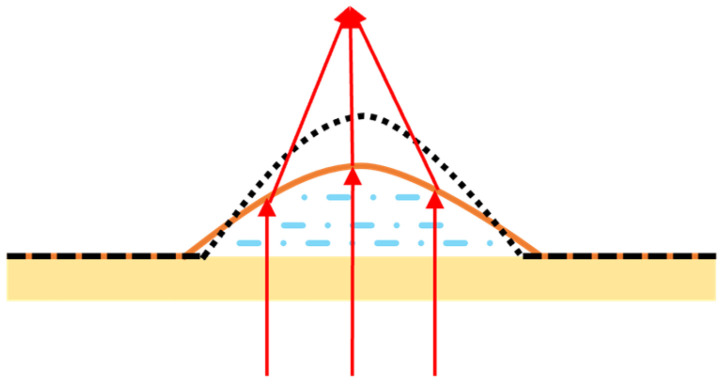
Schematic diagram of MEMS varifocal liquid lenses. Changing the shape of the droplet can convert incident light to different positions.

The principle of MEMS varifocal liquid lenses is very simple, as shown in Figure 17. Changing the shape of the liquid droplet allows incident light to converge. Over the past two decades, many MEMS varifocal liquid lenses have been proposed in both the academic and the industrial communities. Table 4 summarizes MEMS varifocal liquid lenses from relevant references. We roughly divide these devices into five types: electrostatic, electrowetting, dielectrophoretic, piezoelectric, and fluidic.

##### Electrostatic Type

**Figure 18:**
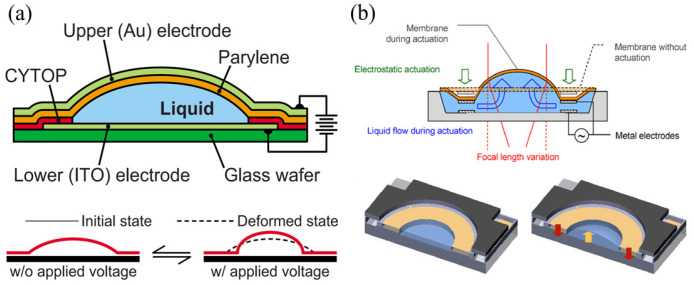
(**a**) Structure and deformation mechanism of a liquid lens packaged in a parylene thin film. Reprinted with permission from Ref. [110]. Copyright 2008 AIP. (**b**) Schematic diagram of the proposed liquid lens, sandwiched between parallel plate capacitors. Reprinted with permission from Ref. [113]. Copyright 2011 ELSEVIER.

Simply put, the electrostatic type combines liquid lenses and MEMS electrostatic actuators. A droplet covered by a thin and transparent film is used as a lens component and sandwiched between two electrodes. When the actuator is operated, a generated electrostatic force pushes the droplet into the center for droplet deformation. Figure 18a shows a design fabricated by Parylene-on-liquid deposition (POLD) [21,105,110]. A silicone oil droplet was dropped onto the indium tin oxide (ITO) bottom electrode patterned on a glass substrate and then encapsulated by a thin Parylene-C film. The device finished after evaporating an Au film as another electrode, which was thin enough for light to pass through. The electrostatic force generated pulled the top electrode toward the bottom electrode, pushing the droplet into the center to change the curvature. The change in curvature caused a focal length variation. They demonstrated that under a voltage of 150 V, the focal length of a 1 mm aperture lens could change to 20% of its initial focal length (3.7 mm). Pouydebasque et al. [113] reported a new type of varifocal liquid lens with an integration of a parallel plate electrostatic actuator, as illustrated in Figure 18b. The liquid lens was fabricated on a glass wafer with a deposited ring-shaped AlSi electrode. A perylene-C film, which functioned as a cover for liquid, was fabricated on another silicon wafer with the same AlSi electrode. By bonding two wafers, the varifocal liquid lens formed. An applied voltage resulted in attraction to move the electrode, which caused the liquid between two electrodes to be pushed to the center. The experimental results showed that such a lens with a diameter of 3 mm could achieve an optical power variation of 8 D.

##### Electrowetting Type

**Figure 19:**
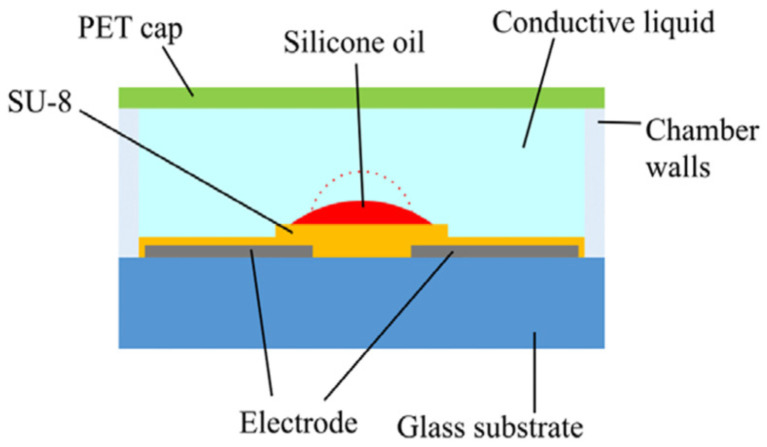
Cross-section of the electro-hydrodynamically actuated liquid lens. Reprinted with permission from Ref. [119]. Copyright 2015 IOP Publishing.

The so-called electrowetting effect refers to the change in contact angle between a liquid and a solid when an electrostatic field is applied. It means that the shape of a droplet can be changed by applying an electrostatic field. Figure 19 shows a lens reported by Ashtiani et al. [119,120]. A layer of area-density-modulated Al electrode was patterned on a glass substrate. A layer of SU-8 was deposited on it as electrostatic isolation. Silicone oil was placed on the SU-8 layer, and the entire chamber was then filled with conductive liquid. The silicone oil droplet was radially pushed inward by applying a voltage to the electrode, resulting in a change in its curvature. The measured focal length varied from 10.1 mm to 5.8 mm as the applied voltage varied from 0 to 100 V. The specially designed electrode could force the droplet to be positioned at the center because at this position the entire system was in the least amount of potential energy. The structure of the electrowetting lens reported by Seo et al. [122] is similar. Conductive liquid droplets were placed on a glass substrate with a patterned ITO electrode on its surface, and the chamber was filled with silicone oil. The electrode comprised of five sections: a grounded central circle electrode and four surrounding fan-shaped electrodes. As observed, focal length variation over 20 mm and focus lateral displacement of 0.346 mm could be obtained.

##### Dielectrophoretic Type

**Figure 20:**
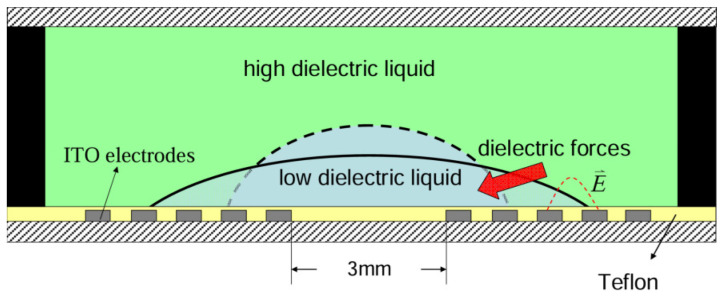
Illustration of the dielectric liquid lens and its varifocal principle. Reprinted with permission from Ref. [106]. Copyright 2007 Optica Publishing Group.

For two immiscible liquids with different dielectric constants, when an electric field is applied, the difference in dielectric constants generates a dielectric force, causing a change in the interface between the two liquids. Cheng et al. [106] introduced a packaged varifocal liquid lens. Figure 20 depicts the structure of such a lens. The lens consisted of a droplet with a low dielectric constant and a sealing liquid with a high dielectric constant. The two liquids were placed in a chamber made of PMMA, and then the whole device was sealed between two glass substrates. The patterned ITO electrode was on the bottom glass substrate. Shrinkage of the droplet could be achieved by exerting a dielectric force onto the droplet. The deformation increased the contact angle between the droplet and the glass surface and thus shortened the focal length. Almoallem et al. [121] reported a lens with a double-sided electrode structure. The structure possessed a pair of electrodes fabricated on a thin Si wafer with a deposited 200 nm thick SiO_2_ layer. The liquid chamber was built using SU-8 while assembling two electrodes. The focal length varied from 67 to 14 mm when the applied voltage was increased from 0 to 25 Vrms, showing the advantage of lower energy consumption.

##### Piezoelectric Type

**Figure 21:**
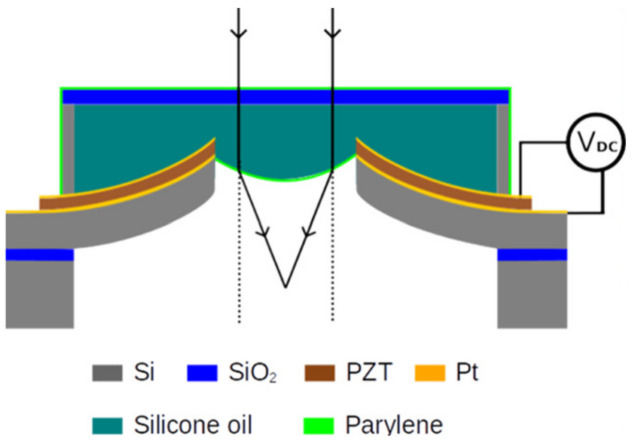
MEMS varifocal liquid lens with PZT actuator and its physical picture. Reprinted with permission from Ref. [125]. Copyright 2024 ELSEVIER.

Piezoelectric actuators can also be used to drive MEMS varifocal liquid lenses for their ability to generate significant force, low energy consumption, and fast speed. Schneider et al. [108,109] used a batch hot press and an oxygen plasma bonding process to fabricate a lens. The lens contained two liquid chambers: a lens chamber filled with liquid and an actuator chamber. When a voltage was applied to the piezoelectric actuator, the liquid in the actuator chamber was pumped into the lens chamber. Due to the entry of new liquid, the lens chamber stretched, changing the curvature of it. As the applied voltage increased from −9 V to 44 V, the focal point of a 5 mm lens moved from 30 to 500 mm. Nicolas et al. [118] proposed a varifocal liquid lens whose structure was similar to the structure in Figure 18b. The main difference is that the electrostatic actuator was replaced with a PZT piezoelectric actuator. With an applied voltage, the mechanical force generated from the deformation of the PZT layer could make the liquid inside the chamber gather inward, causing the curvature to vary. The 3 mm aperture lens could achieve 10 D optical power variation with a wavefront error of less than 50 mm. Tang et al. [124,125] presented a novel piezoelectric MEMS varifocal liquid lens with a thin film actuator, as shown in Figure 21. The actuator was a Si/PZT unimorph disk-shaped structure. It was in direct contact with the liquid from the PZT side or silicon side. When a voltage was applied, the force was generated to bend the actuator up or down. The deformation could cause the liquid to be squeezed, thereby altering the curvature of the lens. Optical measurements indicated that a liquid lens with a diameter of 1.5 mm could change its focal length from infinity to 31.6 mm.

##### Fluidic Type

**Figure 22:**
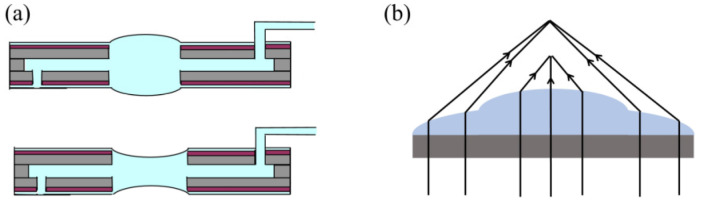
(**a**) MEMS varifocal liquid lens allowing biconvex or biconcave deformation. Reprinted with permission from Ref. [102]. Copyright 2004 IOP publishing. (**b**) Schematic diagram illustrating realization of two distinct focal points.

The fluidic type changes the curvature of the lens by injecting or extracting fluid. This approach is quite simple and easy to implement. Agarwal et al. [102] fabricated a thin PDMS film lens actuated by liquid using a standard photolithography process and silicon-based MEMS technology. The lens allowed biconvex or biconcave deformation by using a micropump to pull in or out the liquid, as presented in Figure 22a. The focus varies from 75.9 mm to 3.1 mm, with the field of view (FOW) changing from 0.12 to 61° under the biconvex state, while under the biconcave state, the focus varies from −75.9 mm to −3.3 mm, with the FOW changing from 7 to 69°. Werber et al. [103] manufactured a varifocal liquid lens covered by a PDMS thin film on a glass substrate. Continuously blowing air into the liquid chamber of the lens exerted a positive pressure of 20 kPa onto the lens, reducing the focal length to 3 mm. They also designed an optical coherence tomography (OCT) system using this lens in the sample arm. Optical experiments proved that the OCT system based on this lens could increase the imaging range while maintaining high lateral resolution. As shown in Figure 22b, Yu et al. [111] reported a liquid lens with two different focus points. The device had two liquid chambers with distinct thicknesses. When pressure was exerted on the chambers, the curvature of the two chambers changed independently, causing incident light to converge at two positions.

Although MEMS varifocal liquid lenses have gained increasing attention due to their diverse types, improved optical performance, and simple principles, the presence of liquid poses considerable challenges to microfabrication.

### 2.4. MEMS Phased Varifocal Mirrors

MEMS phased varifocal mirrors are micromirror arrays composed of numerous tiny mirror elements. As mentioned in Section 2.1.1, the basic principle is to control the phase of the incident light. A driven MEMS phased varifocal mirror is divided into different phase control zones by exerting a specific driving signal to each region. When the incident light wave reflects at different phase control zones, the reflected light waves at different positions interfere with each other, ultimately resulting in the convergence phenomenon. The cyclic variation of the phase control zones allows various focal lengths.

**Figure 23:**
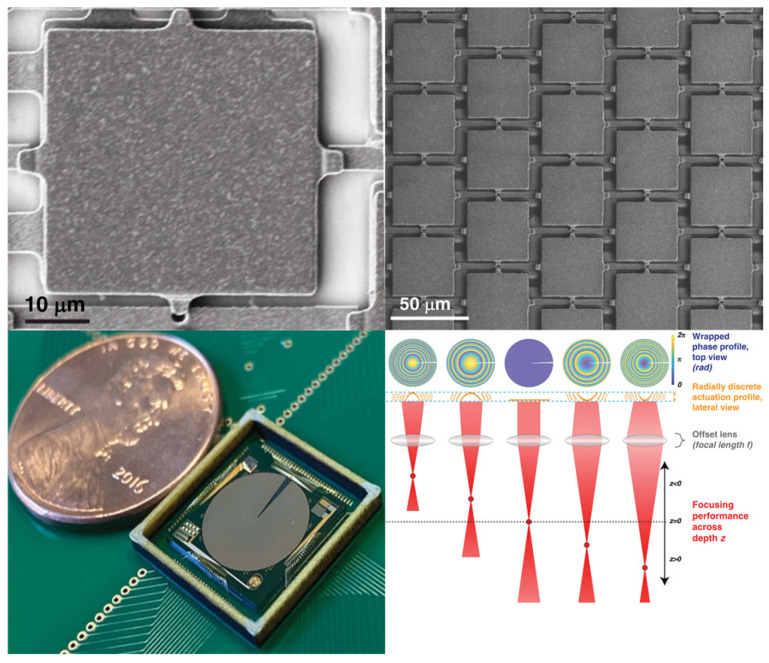
MEMS phased varifocal mirror with annular phase structure. Reprinted with permission from Ref. [129]. Copyright 2020 Nature Publishing Group.

Following conventional micromirror array classification criteria, MEMS phased varifocal mirrors are categorized into two distinct configurations: 1D (linear) arrays and 2D arrays. The typical linear MEMS phased varifocal mirror is proposed by Hamann and his coworkers [127,128]. The device consisted of 1088 electronically addressable elements above a ground electrode. Each element, coated by highly reflective aluminum, was composed of six silicon nitride ribbons. For incident light with a wavelength of λ, a delay phase of 2π can be provided when the component is moved downward by a distance of λ/2. The total axial scan range of 80 mm was realized by using the proposed linear phased array. Landry, Hamann, and Solgaard [128] proposed a random-access cylindrical lensing and beam steering technique using this phased array. By applying cylindrical and linear gradient phase to each element, synchronous axial focal adjustment and lateral beam steering were achieved in a 488 nm laser system. At a numerical aperture (NA) of 0.008, an 8 cm focal shift was achieved with 90% diffraction efficiency and wavefront error below 0.07 waves. When the NA increased to 0.013, the focal point split into multifocal points, while still supporting 350 kHz random-access lateral scanning (±0.55° range). These results show the potential of this MEMS phased array for high-speed dynamic light beam control. Ersumo et al. [129] reported an electrostatic 2D MEMS micromirror array with a diameter of 8.2 mm, consisting of 23,852 square micromirrors as pixels. These pixels were arranged to form a specific portioning geometry, which had a central circle and 31 rings, using an iterative optimization process. Applying discrete voltages to central circles and rings forms discrete annular phase planes, which can provide 0 to 2π phase variation of the incident light, as described in Figure 23. Different focus positions can be obtained by varying the gradient of the phase plane. They also designed a 3D scanning frame using this micromirror array [14].

The principle of MEMS phased varifocal mirrors is not novel. Space Light Modulators (SLMs), optical phased arrays, and other phase-controllable optical devices have already been used to achieve focus tuning. In addition, phase-only spatial light modulators created by TI instruments enable precise phase modulation for focus tuning.

### 2.5. Industrial Commercial Products

Section 2.1, Section 2.2, Section 2.3 and Section 2.4 discussed MEMS varifocal optical elements exclusively within academic research domains. This section emphasizes existing commercialized MEMS varifocal optical elements. A detailed analysis concerning the technical implementation pathways is presented. DigitalOptics Corporation (DOC) pioneered the development of MEMS|CAM modules in the early 2010s, utilizing electrostatic comb-drive MEMS actuators to achieve axial displacement of lens assemblies, thereby replacing conventional voice coil motors (VCMs). The module demonstrates a response speed below 5 ms, achieves power consumption as low as 0.5 mW, and reduces structural height by 33% compared to conventional designs. This design provided compatibility with ultrathin smartphone architectures. Early applications focused on the smartphone sector, having provided test modules for iPhone prototype devices. Subsequently, the application expanded to security surveillance microscale lenses. However, due to insufficient strength to withstand impacts from high-altitude drops, it remained challenging to achieve large-scale commercial deployment.

MEMSDrive introduced a five-axis stabilized autofocus actuator in 2018, utilizing piezoelectric polycrystalline thin-film MEMS technology to drive CMOS sensor displacement. The technology employs the inverse piezoelectric effect to achieve sub-micrometer (0.1 μm) precision displacement of the sensor, with a response time of less than 5 ms, power consumption of merely 1 mW, and a lifetime exceeding 500 million cycles [130]. The core innovation lies in the integration of MEMS actuators with multi-frame super-resolution algorithms, enabling the synthesis of high-resolution images through sensor displacement. The technology has been integrated into Huawei Mate-series periscope lens modules, significantly enhancing telephoto stabilization performance. The implementation has further extended to drone gimbal cameras and AR/VR devices. Compared to DOC solutions, MEMSDrive utilizes piezoelectric materials to overcome driving force limitations, supporting a larger displacement range of ±100 μm, and has become the first MEMS varifocal technology to achieve mass adoption in consumer electronics.

PoLight commercialized the TLens module in 2020, employing STMicroelectronics’ (ST) 8-inch piezoelectric MEMS thin-film process. The technology emulates the human crystalline lens deformation mechanism, achieving millisecond-scale focal adjustment through voltage-controlled curvature modulation in polymer optical elements. The device features a thickness of merely 0.45 mm, a response time of 1 ms, and power consumption of only 0.1 mW [131]. In 2021, TLens achieved initial mass production deployment in the lateral camera module of Xiaomi Mitu Kids Watch 4 Pro (produced by Xiaomi Corporation, Beijing, China), addressing autofocus challenges inherent in wearable imaging systems. The application has since expanded to AR glasses (e.g., Vuzix Blade), medical endoscopes, smartphones, autofocus webcams, and so on. TLens’s liquid lens solution avoids the traditional mechanical displacement limitations and is currently the thinnest commercial MEMS varifocal module.

Overall, there are two main approaches for commercial MEMS varifocal technologies: one is replacing voice coil motors (VCMs) with MEMS actuators to move optical elements, and the other employs active optical elements. Moreover, commercial applications primarily focus on various imaging systems, such as smartphone camera modules, micro cameras, AR/VR head-mounted displays, drone cameras, and others. Compared to commercial varifocal technologies, MEMS varifocal optical elements proposed in academic research can provide faster and larger-range focus control. For imaging systems, the use of MEMS reflective varifocal mirrors or MEMS phased varifocal mirrors can reduce spherical aberration, enhancing imaging quality. Finally, MEMS varifocal optical elements from academic research have demonstrated applicability across diverse optical systems, including 3D imaging arrays, laser systems, high-resolution microscopies, precision optical measurements.

## 3. Applications

MEMS optical varifocal elements have the potential to be used in various optical systems. The following content provides a detailed introduction to the current application areas.

### 3.1. Confocal Optical Instruments

**Figure 24:**
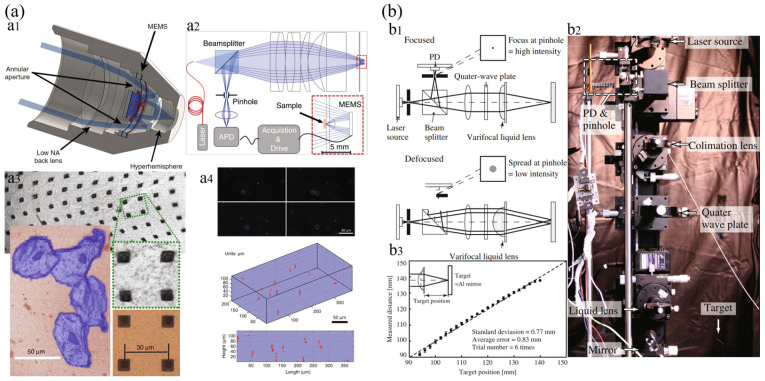
(**a**) Confocal microscopy system with MEMS-in-the-lens architecture. (**a1**,**a2**) shows the miniaturized confocal microscope incorporating a MEMS 3D scanner and the confocal imaging system utilizing the miniaturized microscope. (**a3**) is the experimental imaging results on the surface of a prototype three-dimensional scanner and human cheek cells. The upper 4 figures of (**a4**) are images of 6 µm diameter polystyrene beads suspended in ultrasound gel at different focal length. The bottom two figures are the reconstruction of three-dimensional distribution through the images recorded at each focal plane. Reprinted with permission from Ref. [17]. Copyright 2019 Nature Publishing Group. (**b**) The confocal distance sensor uses a varifocal liquid lens. (**b1**) is the diagram about how to measure distance using the confocal optical system. (**b2**) is the physical picture of the designed confocal optical system. (**b3**) is analysis of the deviation between the target distance and the actual measured distance. Reprinted with permission from Ref. [21]. Copyright 2014 Springer Berlin Heidelberg.

Confocal optical instruments utilize confocal microscopy technology to achieve high-precision imaging or precise measurement. High-quality optical imaging or high-precision optical measurement can be realized by scanning the sample laterally and axially while using a small hole to block defocused backscattered light [132]. A series of confocal optical instruments, including confocal microscopes, confocal displacement sensors, and confocal profilometers have been designed and implemented in many fields such as biological tissue imaging, clinical medical diagnosis, industrial measurement. Liu et al. [16,17,38] reported a confocal microscopy system with MEMS-in-the-lens architecture, where a MEMS reflective mirror was placed between two lenses, as shown in Figure 24a. The prototype successfully imaged a three-dimensional scanner, human cheek cells, and 6 µm diameter polystyrene beads suspended in ultrasound gel. Moghimi et al. [18] described a confocal microscope utilizing an electrostatic MEMS reflective varifocal mirror. Living arrowhead Syngonium leaf tissue had been imaged to demonstrate the practicality of the proposed confocal microscope. Noda et al. [21] developed a confocal distance sensor using a MEMS varifocal liquid lens. The liquid lens was deformed at 0.2 Hz. They demonstrated that the confocal distance sensor could measure a distance of 94 to 140 mm with an average error of 0.83 mm. The results are depicted in Figure 24b. However, the sampling frequency of 0.2 Hz cannot meet the requirements of commercial applications where the sampling frequency can reach 2 kHz. In Ref. [22], Nakazawa et al. reported a novel confocal displacement sensor using a MEMS reflective varifocal mirror. Its working distance was 31 mm. The system could achieve a displacement measurement of 310 μm at a sampling frequency of 7 kHz, while maintaining an absolute linear error of less than 1.2%. At the same time, the research group used the sensor to measure the surface profile of a grooved glass plate. The deviation between the measured profile and the ideal profile was maintained within a range of 3 μm.

### 3.2. Optical Coherence Tomography (OCT)

**Figure 25:**
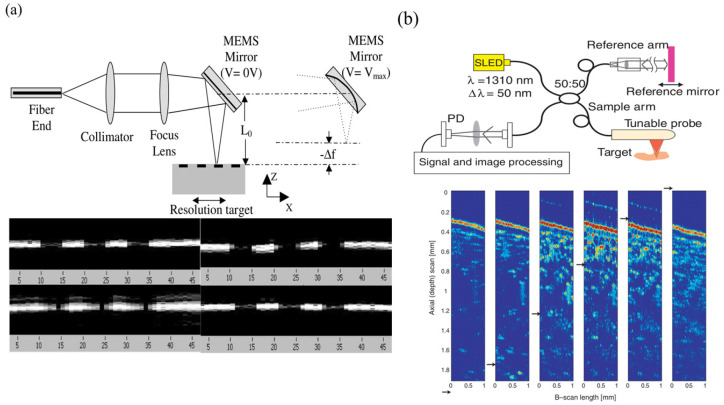
OCT systems. (**a**) The upper figure shows the high-speed OCT system uses an elliptical MEMS reflective varifocal mirror. The bottom are images of the 50-lm bars on a USAF resolution target. On the left side, the MEMS reflective varifocal mirror is used as a simple reflector, while on the right side, it is employed for varifocal purpose. Reprinted with permission from Ref. [32]. Copyright 2004 Elsevier. (**b**) Time-domain optical coherence tomography (OCT) system using a pneumatically driven MEMS varifocal liquid lens. The figure on the upper right corner shows the configuration of OCT system with scanning reference arm and sample arm. The figure on the bottom right corner is derived from six B-scan steps on the same section of an onion slide for different focal lengths. The arrows approximately indicate position of the focal point. Reprinted with permission from Ref. [19]. Copyright 2008 IOP Publishing.

Optical Coherence Tomography (OCT) is a non-contact optical imaging technique widely used in biological tissue imaging, retinal examination in clinical diagnosis, and skin cancer detection. The basic principle of OCT is similar to ultrasound imaging technology, except that light is employed in OCT systems instead of acoustic wave. Low coherent light is guided to the sample (sample arm) and the reference object (reference arm). The intensity and the delay of the back-reflected light from the sample arm are analyzed to reveal the depth where the reflection occurred [133]. The analyzation is obtained through interference measurement of light reflected from the sample arm and reference arm. Combined with lateral scanning, high-resolution 3D imaging can be achieved. As shown in Figure 25a, Qi et al. [32] represented a high-speed OCT system using an elliptical MEMS reflective varifocal mirror. The feasibility of operation synchronous with a 8 kHz OCT depth scan frequency was demonstrated. The lateral resolution at a distance of 1 mm from the focus position was consistent with the lateral resolution at the focal point, suggesting that the system improved the lateral resolution while maintaining a large imaging depth. They also theoretically proved that the varifocal mirror with a large stroke can allow application in high numerical aperture imaging systems [53]. Yang et al. [33] designed a Doppler OCT imaging system based on a MEMS reflective varifocal mirror. By imaging and analyzing the 0.5% lipid suspension and flowing 0.5% intralipid solution, it was demonstrated that the imaging quality and Doppler shift estimation accuracy of the OCT systems were improved. Aljasem et al. [19] integrated a novel miniaturized fiber optic tunable endoscopic probe using a pneumatically driven MEMS varifocal liquid lens into a time-domain optical coherence tomography (OCT) system, as illustrated in Figure 25b. To demonstrate the functionality of the OCT system, a test structure consisting of eight cover glass slides was imaged. As the focus of the system changed, the position of peak intensity also constantly shifted. A 3 mm thick slice of onion skin was imaged, indicating the 3D imaging ability of the system.

### 3.3. AR/VR

**Figure 26:**
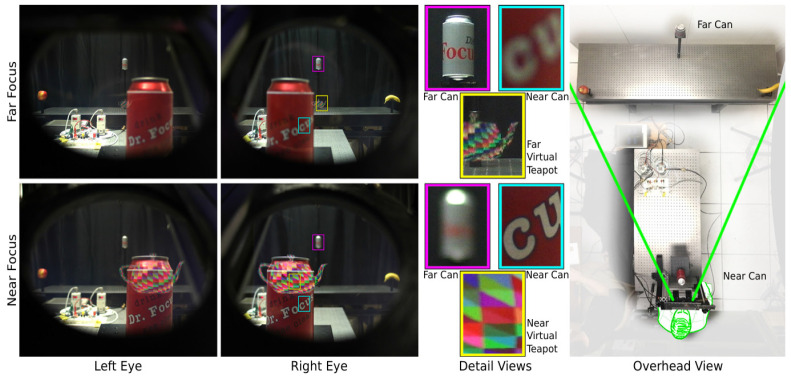
Augmented reality display utilizing a transparent thin-film deformable mirror shows a virtual teapot at near and far distances together with two cans. Reprinted with permission from Ref. [15]. Copyright 2017 IEEE.

AR (Augmented Reality) is the technology that projects virtual images through a camera or video viewer into the real world. On the other hand, VR (Virtual Reality) is the technology that takes these virtual images to create an entirely simulated alternate world. Since the proposal of AR and VR, they have been highly regarded by researchers and technology companies around the world. The practical applications of AR and VR have brought revolutionary changes to our lives, such as education, healthcare, industrial training, movies, and games. With the emergence of the metaverse, a global boom of AR and VR has erupted once again. However, Vergence Accommodation Conflict (VAC) deeply limits their practical application [134]. When a user wears an AR/VR headset, the crystalline lens of each eye is naturally changed to focus on the display screen while two eyes converge on the image. Because the distance from the screen to the eyes is always different from that from the image to the eyes, vergence distance cannot match focus distance. This fact causes dizziness and vomiting [135]. One way to solve VAC is to use varifocal technology to match vergence distance and focus distance. As shown in Figure 26, Dann et al. [15] used a transparent thin film deformable mirror that can achieve a tuning range of 14 D to manufacture an AR near display device. The device could not only solve VAC but also permit a wide field of view (100° diagonal).

### 3.4. Laser Applications

As a novel light source characterized by monochromaticity, directionality, and high power, lasers have been extensively utilized in various modern optical systems. The application of MEMS varifocal optical elements provides new possibilities for portability and miniaturization in various laser systems. Lukes and Dickensheets [25] used an elliptical MEMS deformable mirror driven by three independent electrodes to create a laser read-write head. The mirror is integrated at a 45-degree angle of incidence into an optical system composed of a DVD objective lens (NA = 0.6) and a HeNe laser (633 nm). The experimental results proved that the varifocal mirror supports wide-range focus switching, such as accommodating the 46.5 μm interlayer thickness variation in BDXL™ discs. By utilizing three-electrode actuation for mirror deformation, a wavefront aberration compensation capability of 1.6 μm was achieved at 7.5 μm central displacement, successfully suppressing residual spherical aberration in quadruple-layer BDXL™ discs below 130 nm. The proposed mirror exhibited significant potential for laser read-write heads with both focus control and spherical aberration correction capabilities.

Kopf et al. [24] proposed a unimorph varifocal mirror that integrates a copper heat dissipation layer. When using a 6.3 kW high-power laser as the test light source, the mirror achieved a 3.6 mm focal shift under 600 V driving voltage, with a response time under 2 ms. The system maintained a wavefront RMS deviation of ±42.6 nm and a beam quality factor M^2^ stabilized at 3.0. This provides a high-speed and high-thermal-stability focal control solution for high-power three-dimensional laser cutting, welding and other applications. Geraldes et al. [20] developed an auto-focusing laser system based on a varifocal mirror using hydraulic actuation. Addressing the issue of tissue carbonization in endoscopic laser surgery, where the fiber tip is often placed in direct contact with tissues instead of utilizing laser focusing for cutting, this system provides a novel surgical tool. Experimental validation confirmed the system’s capability to maintain laser spot diameter fluctuation within 3% across a 12.15–52.15 mm working range. This proved the feasibility of MEMS varifocal mirrors in the medical field. Rabczuk and Sawczak [23] proposed a method to optimize output characteristics of high-power continuous-wave CO_2_ lasers through dynamic adjustment of optical cavity configurations, utilizing coolant pressure-controlled variable-curvature mirrors. The experimental results demonstrate that tuning the radius of curvature (R) of the rear mirror significantly impacts laser performance. As R increases from 3.4 m to 10.5 m, the output power initially rises to a peak of 600 W at R = 5 m, then declines due to increased diffraction losses. The beam quality factor M^2^ reaches a minimum of 1.6 (near-fundamental TEM_00_ mode) at R = 6 m but increases to 2.4 for R < 4 m or R > 8 m due to higher-order mode mixing. Intensity profiles reveal that curvature adjustments enable transitions between multimode mixtures and dominant fundamental modes. The study confirms that dynamic curvature control (range: infinite to 3 m) allows flexible tuning of power and mode characteristics while maintaining beam quality comparable to conventional fixed-curvature mirrors, offering a viable adaptive solution for industrial high-power lasers.

### 3.5. Optical Communication

Optical communication is a technology that utilizes light waves for information transmission, characterized by high speed, broad bandwidth, strong resistance to electromagnetic interference, and suitability for long-distance transmission. A typical optical communication system comprises essential components, including a light source, optical fiber, optical receiver, modulator, optical filter, and optical switch. This technology has found extensive applications in data centers, 5G or 6G networks, LiDAR systems, and medical imaging domains. Notably, MEMS have emerged as a pivotal driving force for advancing optical communication technologies due to their miniaturization capabilities, low power consumption, and rapid response characteristics. In the context of explosive growth in computational power demands and the pervasive integration of AI technologies, the deep combination between MEMS and optical communication can accelerate the development of more efficient and intelligent optical network architectures. Pollock et al. [41] proposed a thermally driven MEMS reflective varifocal mirror, which can realize lateral and axial light steering through tip-tilt motion. It features a ±40° tilt angle variation and an adjustable curvature radius range from −0.48 mm to 20.5 mm. By positioning the MEMS varifocal mirror at a distance of 1.275 mm from the single-mode optical fiber and applying an electrical power of 12.9 mW, a minimum beam divergence angle of 0.18° and a beam diameter of 18 mm at 3 m (approaching the diffraction limit) were achieved. This demonstrates that the communication system utilizing this MEMS varifocal mirror exhibits significantly superior long-range propagation performance compared to direct fiber output and static collimation systems. They established a bit error rate (BER) test system. With the fiber-to-mirror distance fixed at 1.275 mm, electrical power applied to the varifocal mirror was adjusted to modify the beam diameter. At 12.9 mW power, the beam achieved a focusing diameter with BER < 10^−9^. Maintaining static detector position, the lateral displacement of the beam center was measured via mirror tilting. BER increased from 10^−9^ to 10^−6^ at 0.5 mm beam-detector misalignment. These demonstrate the MEMS varifocal mirror’s capability for high-SNR communication and dynamic tracking of mobile terminals.

### 3.6. Adaptive Optical Zoom

Optical zoom is crucial for optical imaging systems such as cameras, camcorders, surveillance cameras, and smartphone cameras. These imaging systems vary the magnification or image objects at different distances by changing the focus length. Conventional technology uses zoom systems that consist of multiple moving lenses [136]. Replacing moving optical elements with active optical elements significantly reduces the size of the optical system and improves response time. Kaylor et al. [11] designed a small non-mechanical zoom camera with a volume of 3 cm × 3 cm × 1.7 cm, featuring a 15° field of view and achieving a magnification of 15×. Hsieh et al. [12] manufactured a self-focusing camera module with a volume of only 1 cm × 1.15 cm × 0.67 cm, achieving a f-number of 4.13 and an on-axis MTF of 0.28. Meanwhile, it had been demonstrated that this module could provide clear images of objects at different positions. Ref. [13] presents a micromachined fluoropolymer deformable mirror designed by Wang and his coworkers. The imaging experiment results showed that the designed auto-focusing system could provide clear imaging of objects at different distances and proved the possibility of use in mobile electronic devices.

## 4. Comparison

This section provides comprehensive comparisons of MEMS varifocal optical elements. As mentioned in Section 2.1.3, I, as a performance indicator that can comprehensively characterize axial resolution and lateral resolution, is used as the main reference for comparisons.

### 4.1. Comparison of Non-Resonant MEMS Reflective Varifocal Mirrors

**Figure 27:**
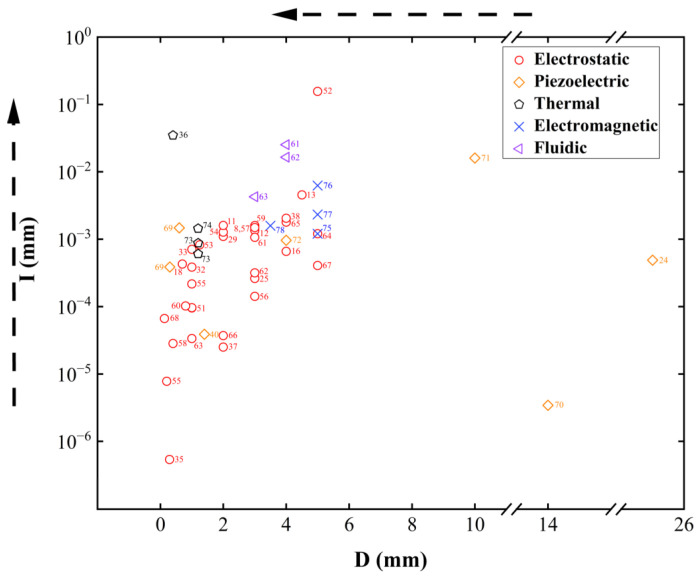
Comparison of five types of non-resonant MEMS reflective varifocal mirrors. Numbers near markers on the plot correspond with references.

Figure 27 shows a comparison of five types of MEMS reflective varifocal mirrors. The y-axis is I, and the x-axis is the effective diameter. The closer the performance indicator is to the top left corner of the image, the greater the potential to achieve high-quality imaging and promote miniaturization. Electrostatic actuation is the most important and mature actuation method, which can ensure high imaging quality and meet the aperture requirements of numerous optical systems (1 to 6 mm). Utilizing piezoelectric, fluidic, and thermal actuation can surpass the performance of most electrostatic types. However, considering that heat can easily cause damage to optical systems, thermal actuation is not a very appropriate approach. In addition, utilizing temperature variation to drive is also a drawback for response speed. Due to the presence of fluid, it brings considerable difficulties to the integration with microelectronic devices. The slow response speed is another main reason that limits fluidic actuation. Therefore, piezoelectric actuation has attracted the attention of researchers around the world. The performance of electromagnetic mirrors is not significantly different from that of electrostatic mirrors. However, the electromagnetic types typically occupy more space for the necessary size limitation of electromagnetic actuators. The improvement of response speed also must be carefully considered. Table 5 summarizes the characteristics of these five types.

### 4.2. Comparison of Resonant MEMS Reflective Varifocal Mirrors

**Figure 28:**
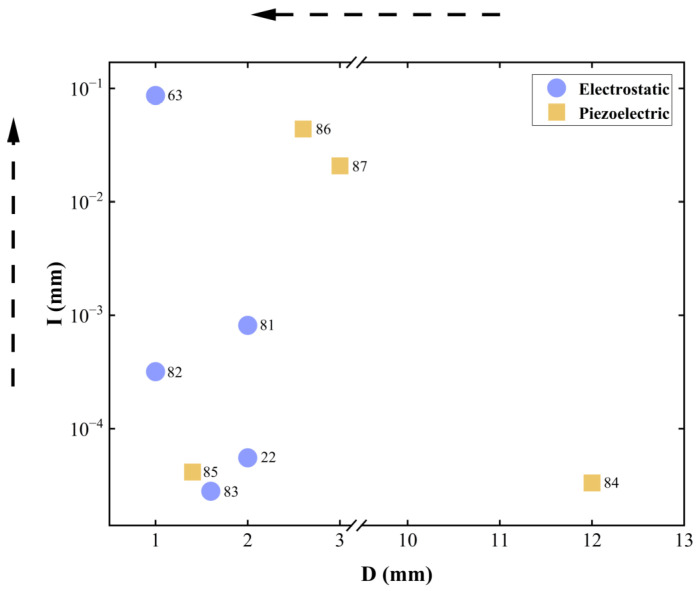
Comparison of resonant MEMS reflective varifocal mirrors using *I*. Numbers near markers on the plot correspond with references.

A comparison of MEMS reflective varifocal mirrors used at resonant mode is shown in Figure 28. Both electrostatic and piezoelectric mirrors can significantly improve the performance of varifocal mirrors compared to non-resonant conditions when the same voltage is applied.

**Figure 29:**
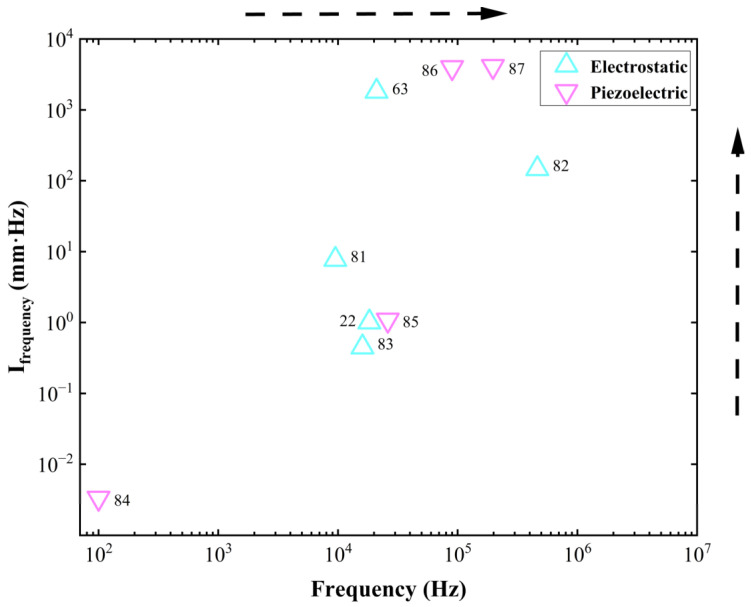
Comparison of resonant MEMS reflective varifocal mirrors using $I_{frequency}$. Numbers near markers on the plot correspond with references.

Figure 29 describes the relationship between $I_{\mathrm{frequency}}$ and $f_{resonance}$. It should be noted that the performance indicator $I_{\mathrm{frequency}}$ is a product of *I* and $f_{resonance}$, reflecting the varifocal speed of this type. The speed of the mirror can reach up to 1 μs (1 MHz). The minimum is not less than 1 ms (1 kHz). The performance of piezoelectric actuation and electrostatic actuation is very similar, but we believe that piezoelectric mirrors are more attractive because it offers unrestricted movements and boasts exceptional mechanical properties. The resonant frequency is largely determined by the material, so using high-frequency piezoelectric materials can enable fast varifocal speed.

### 4.3. Comparison of MEMS Varifocal Microlenses

**Figure 30:**
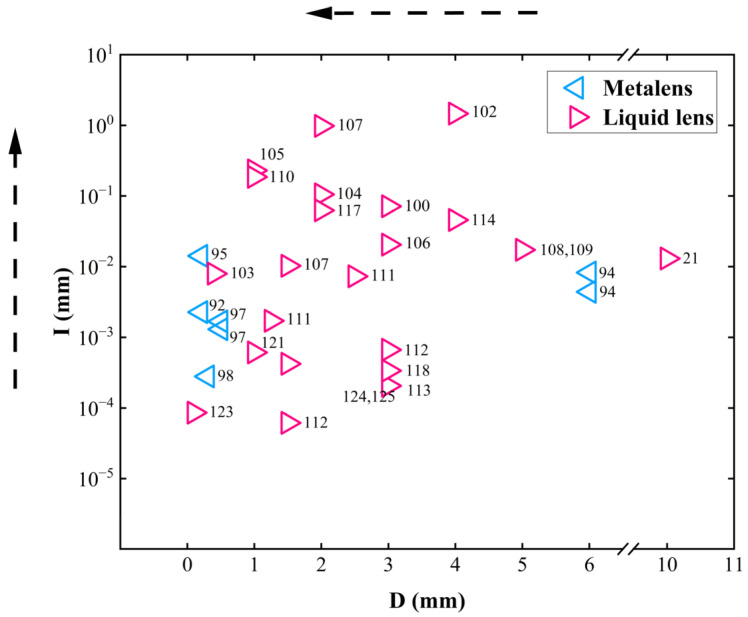
Comparison of MEMS varifocal microlenses. Numbers near markers on the plot correspond with references.

The performance of MEMS varifocal microlenses is depicted in Figure 30. MEMS varifocal metalenses perform well in small sizes, proving their enormous potential. The performance of MEMS varifocal liquid lenses is relatively dispersed. Compared with MEMS reflective varifocal mirrors, an interesting phenomenon is that indicator I of MEMS varifocal microlenses generally has an order of magnitude larger can be observed. By contrasting the focal length variation of the two types, the fact that MEMS varifocal microlenses always have a smaller minimum focal length can explain this condition. However, MEMS reflective varifocal mirrors generally have a maximum focal length reaching infinity as the initial mirror surface is always flat and without deformation. MEMS varifocal microlenses, particularly liquid lenses, always have a spherical droplet with variable curvature. Hence, some devices cannot support an infinity focus position.

### 4.4. Overall Comparison

We present an overall comparison among the three types of MEMS varifocal optical elements in Table 6. Three conclusions can be drawn from the comparison figures and tables: (1) In the past twenty years, MEMS varifocal optical elements based on various principles have emerged one after another. This is a field full of opportunities, attracting more and more research interests. (2) Among the three types of MEMS varifocal optical elements, MEMS reflective varifocal mirrors are the most mature technology. Further studies on the other two types are needed. Some microscale physical phenomena also need to be clarified. (3) Most MEMS varifocal optical elements are still in the laboratory. In the future, the exploration of more applications is also worth paying attention to.

## 5. Conclusions and Outlook

We review MEMS varifocal optical elements in the past two decades and provide a detailed description of classification, the principle of focus control, and practical applications. In addition, comprehensive comparisons of these elements are presented. We anticipate four major development trends emerging in the coming years. Firstly, light field manipulation provides a new feasible technical solution for MEMS varifocal technology. The implementation of this approach can be achieved either by developing dedicated optical elements like MEMS-based phased varifocal mirrors and varifocal metalenses, or by utilizing existing light-field modulation devices such as phase-only spatial light modulators and digital micromirror devices (DMD). Light field manipulation makes full use of the characteristic of MEMS’s small size, enabling faster focus control. The principle of phase control can remove restrictions caused by mechanical structure to the varifocal range. Secondly, utilizing materials such as piezoelectric materials, polymer materials, and metasurface materials can improve the performance. Piezoelectric materials are used in actuators to provide rapid response and significant driving force. Additionally, the resonant characteristics make piezoelectric materials more suitable for high-speed dynamic focal scanning. Polymer materials are utilized to construct the structures of MEMS varifocal optical elements, such as the support structures for reflectors or the thin films of lenses. The low-stress, soft, and large-deformation characteristics of polymers significantly improve the performance of MEMS varifocal optical elements. The artificially fabricated metamaterials have demonstrated the possibility of planar varifocal optical elements. Its introduction has provided MEMS varifocal technology with a much broader range of material options as well as better optical performance. Furthermore, the current mature applications are mainly focused on imaging systems, with lens-based products dominating commercialization efforts. Future research directions can focus on applications such as optical scanning, precise optical measurement, and light control for optogenetics, among others.

With the increasing demand for miniaturization and high integration of optical systems, as well as the continuous development of electronic technology, we believe that MEMS varifocal optical elements will be widely used in many fields, with mature commercial applications emerging at an explosive pace.

## Figures and Tables

**Table 1:** Summary of non-resonant MEMS reflective varifocal mirrors.

Type	Year	Author	Deformation	Mirror Size	Focal Length Variation	*I* (×10^−6^)
Electrostatic	2001	Himmer et al. [29]	concave	1 mm × 1 mm	36 mm–360 mm	95.49 mm
	2003	Himmer et al. [51]	concave	1.4 mm × 1 mm *	36 mm–inf	96.45 mm
	2004	Qi et al. [32]	concave	1.4 mm × 1 mm *	18 mm–inf	385.8 mm
	2004	Shao et al. [34]	concave	0.7 mm × 0.7 mm	10 mm–inf	428.75 mm
	2006	Mescheder et al. [52]	concave	5 mm × 5 mm	10 mm–inf	156,250 mm
	2006	Dickensheets et al. [53]	concave	1.25 mm × 1.25mm	17.2 mm–inf	825.25 mm
	2006	Yang et al. [33]	concave	1.4 mm × 1 mm *	13.3 mm–inf	706.65 mm
	2009	Lukes et al. [54]	concave	2 mm × 2 mm	30.1 mm–inf	1103.74 mm
	2009	Hokari et al. [55]	convex	1 mm × 1 mm	−24 mm–inf	217.01 mm
				0.4 mm × 1 mm ^#^	−32 mm–−710 mm	7.80 mm
	2009	Wang et al. [13]	concave	4.5 mm × 4.5 mm	50 mm–inf	4556.25 mm
	2010	Hsieh et al. [12]	concave	3 mm × 3 mm	50 mm–inf	1350 mm
	2010	Kaylor et al. [56]	concave	3 mm × 3 mm	154 mm–inf	142.31 mm
	2010	Lutzenburger et al. [57]	concave	3 mm × 3 mm	47 mm–inf	1527.84 mm
	2011	Moghimi et al. [8]	concave	3 mm × 3 mm	47 mm–inf	1527.84 mm
	2011	Sasaki et al. [58]	concave convex	0.4 mm × 0.4 mm	−28 mm–inf 21 mm–inf	28.34 mm
	2011	Lukes et al. [59]	concave	3 mm × 4.24 mm *	47.6 mm–inf	1489.57 mm
	2012	Lukes et al. [25]	concave	3 mm × 4.24 mm *	113.6 mm–inf	261.53 mm
	2012	Moghimi et al. [18]	concave	2 mm × 2mm	28 mm–inf	1275.51 mm
	2012	Sasaki et al. [35]	concave convex	0.29 mm × 0.29 mm	−128 mm–inf 93 mm–inf	0.54 mm
	2012	Kaylor et al. [11]	concave	2 mm × 2 mm	25 mm–inf	1600 mm
	2013	Strathman [60]	concave	0.8 mm × 0.8 mm	25 mm–inf	102.4 mm
	2013	Moghimi et al. [61]	concave	3 mm × 3 mm	56.25 mm–inf	1066.67 mm
	2013	Lukes et al. [62]	concave	3 mm × 4.24 mm *	103.2 mm–inf	316.89 mm
				3 mm × 4.24 mm *	–	–
	2013	Sasaki et al. [63]	concave	1 mm × 1 mm	59.5 mm–271.7 mm	33.61 mm
	2014	Moghimi et al. [64]	concave convex	5 mm × 5 mm	−208.3 mm–inf 135.9 mm–inf	1206.14 mm
	2016	Lukes et al. [65]	concave	4 mm × 4 mm	66.7 mm–inf	1798.2 mm
	2016	Liu et al. [38]	concave	4 mm × 4 mm	62.5 mm–inf	2048 mm
	2017	Nakazawa et al. [37]	concave convex	2 mm × 2 mm	−523 mm–inf 216 mm–inf	25.09 mm
	2018	Liu et al. [16]	concave	4 mm × 4 mm	110 mm–inf	661.16 mm
	2021	Kallmann et al. [66]	concave	8 mm × 2 mm	163.9 mm–3333.3 mm	37.14 mm
	2021	Mescheder et al. [67]	concave	5 mm × 5 mm	196 mm–inf	406.73 mm
	2022	Wang et al. [68]	concave	0.13 mm × 0.13 mm	2.03 mm–79.8 mm	66.6 mm
Piezoelectric	2002	Mescher et al. [69]	concave	0.6 mm × 0.6 mm	1.62 mm–1.75 mm	1471.74 mm
				0.3 mm × 0.3 mm	1.78 mm–2.23 mm	386.53 mm
	2013	Stürmer et al. [70]	concave	14 mm × 14 mm	10,000 mm–inf	3.43 mm
	2017	Kopf et al. [24]	convex	25 mm × 25 mm	−2000 mm–inf	488.28 mm
	2018	Wapler et al. [71]	concave convex	10 mm × 10 mm	−125 mm–inf 125 mm–inf	16,000 mm
	2019	Inagaki et al. [72]	concave convex	4 mm × 4 mm	−120 mm–inf 140 mm–inf	963.72 mm
	2024	Sasaki et al. [40]	concave	1.4 mm × 1.4 mm	85 mm–200 mm	38.90 mm
Thermal	2012	Li et al. [73]	concave	1.2 mm × 1.2 mm	11.5 mm–16.9 mm	876.99 mm
					11.5 mm–14.5 mm	605.92 mm
	2014	Paterson et al. [74]	concave	1.2 mm × 1.2 mm	9.6 mm–15.5 mm	1444.69 mm
	2015	Morrison et al. [36]	concave convex	0.4 mm × 0.4 mm	−0.48 mm–inf 20.5 mm–inf	34,741.26 mm
Electromagnetic	2011	Hashizume et al. [75]	concave	5 mm × 5 mm	111.6 mm–558 mm	1204.4 mm
	2015	Hossain et al. [76]	convex	5 mm × 5 mm	−50 mm–inf	6250 mm
	2015	Hossain et al. [77]	convex	5 mm × 5 mm	−82 mm–−4938 mm	2323.12 mm
	2017	Hossain et al. [78]	convex	3.5 mm × 3.5 mm	−58.1 mm–inf	1587.68 mm
Fluidic	2018	Geraldes et al. [79]	concave convex	4 mm × 4 mm	24.61 mm–inf −25.72 mm–inf	25,302.29 mm
	2018	Geraldes et al. [80]	concave convex	4 mm × 4 mm	32.15 mm–inf −30.26 mm–inf	16,476.56 mm
	2019	Geraldes et al. [20]	concave convex	3 mm × 4.24 mm	−38.3 mm–inf 41.5 mm–inf	4260.43 mm

Tips: In the Mirror Size column, * indicates that the shape of the mirror is elliptical. ^#^ represents rectangle. The data without any annotations indicates that the shape of the mirror is circular. In the Focal Length Variation column, inf represents the infinite focal length.

**Table 2:** Summary of resonant MEMS reflective varifocal mirrors.

Year	Author	Type	Deformation	Mirror Size	Focal Length Variation	Frequency	*I*_resonance_
2013	Sasaki et al. [63]	Electrostatic	concave	1 mm × 1 mm	1.2 mm–271.7mm	21 kHz	1822.88
2016	Nakazawa et al. [81]	Electrostatic	concave convex	2mm × 2 mm	35 mm–inf −41 mm–inf	9.5 kHz	7.76
2017	Nakazawa et al. [22]	Electrostatic	concave convex	2 mm × 2 mm	139 mm–inf −523 mm–inf	18.3 kHz	1.01
2020	Sasaki et al. [82]	Electrostatic	concave convex	1 mm × 1 mm	28 mm–inf −28 mm–inf	462.7 kHz	147.54
2022	Kocer et al. [83]	Electrostatic	concave	1.6 mm × 1.6 mm	110 mm–190 mm	16 kHz	0.45
2006	Tanaka et al. [84]	Piezoelectric	convex	12 mm × 12 mm	900 mm–inf	0.1 kHz	0.003
2018	Janin et al. [85]	Piezoelectric	concave convex	1.4 mm × 1.4 mm	96.2 mm–inf −278 mm–inf	26.1 kHz	1.08
					–	107 kHz	–
2021	Pribošek et al. [86]	Piezoelectric	concave convex	2.6 mm × 2.6 mm	−10 mm–inf 10 mm–inf	90 kHz	3954.6
2023	Pribošek et al. [87]	Piezoelectric	concave convex	3 mm × 3 mm	18 mm–inf −18 mm–inf	197 kHz	4104.2

Tips: In the Mirror Size column, the data without any annotations indicates that the shape of the mirror is circular. In the Focal Length Variation column, inf represents the infinite focal length. inf represents the infinite focal length.

**Table 3:** Summary of major MEMS varifocal metalenses.

Year	Author	Size	Focal Length Variation	I (×10^−6^)
2016	Kamali et al. [92]	0.2 mm × 0.2 mm ^#^	0.6 mm–1.4 mm	2267.57 mm
2018	Arbabi et al. [93]	–	0.627 mm–0.824 mm	–
2018	She et al. [94]	6 mm × 6 mm	50 mm–65 mm	4409.47 mm
			50 mm–103.5 mm	8279.52 mm
2020	Han et al. [95]	0.2 mm × 0.2 mm ^#^	0.182 mm–0.25 mm	14,189.59 mm
2022	Han et al. [96]	0.5 mm × 0.5 mm ^#^	–	–
2022	Han et al. [97]	0.5 mm × 0.5 mm ^#^	2.7 mm–5.8 mm	1678.87 mm
		0.5 mm × 0.5 mm ^#^	3 mm–6 mm	1302.08 mm
2022	Dirdal et al. [98]	0.3 mm × 0.3 mm	1.7 mm–1.95 mm	280.25 mm
2024	Dullo et al. [99]	1.5 mm × 1.5 mm	–	–

Tips: In the Size column, ^#^ represents rectangle and the data without any annotations indicates that the shape of the mirror is circular. In the Focal Length Variation column, inf represents the infinite focal length.

**Table 4:** Summary of MEMS varifocal liquid lenses.

Year	Author	Size	Focal Length Variation	I (×10^−6^)
2003	Krupenkin et al. [100]	3 mm × 3 mm	3.8 mm–4.56 mm	71,416.2 mm
2003	Chronis et al. [101]	–	1.8 mm–6 mm (Norland 63)	–
			0.6 mm–2 mm (Oil)	–
2004	Agarwal et al. [102]	4 mm × 4 mm	−75.9 mm–−3.3 mm 3.1 mm–75.9 mm	1,466,460.45 mm
2005	Werber et al. [103]	0.4 mm × 0.4 mm	1 mm–18 mm	7975.31 mm
2006	Moran et al. [104]	2 mm × 2 mm	2.86 mm–7.69 mm	105,345.22 mm
2007	Nguyen et al. [105]	1 mm × 1 mm	0.8 mm–3.8 mm	186,655.99 mm
2007	Cheng et al. [106]	3 mm × 3 mm	12 mm–34 mm	20,517.95 mm
2007	Aljasem et al. [107]	1.5 mm × 1.5 mm	5 mm–8 mm	10,283.2 mm
		2 mm × 2 mm	1 mm–7 mm	979,591.84 mm
2008	Schneider et al. [108,109]	5 mm × 5 mm	30 mm–500 mm	17,298.61 mm
2008	Binh-Khiem et al. [110]	1 mm × 1 mm	0.8 mm–3.7 mm	186,181.75 mm
2009	Yu et al. [111]	1.25 mm × 1.25 mm	11.95 mm–inf	1709.64 mm
		2.5 mm × 2.5 mm	16.35 mm–inf	7306.25 mm
2011	Pouydebasque et al. [112]	1.5 mm × 1.5 mm	−200 mm–inf 90.9 mm–inf	61.6 mm
		3 mm × 3 mm	−1000 mm–inf 71.4 mm–inf	665.4 mm
2011	Pouydebasque et al. [113]	3 mm × 3 mm	−200 mm–inf 166.7 mm–inf	205.83 mm
2012	Li et al. [114]	4 mm × 4 mm	−15 mm–inf 28 mm–inf	45,759.64 mm
2013	Ashtiani et al. [115]	–	−22.6 mm–inf 22.6 mm–inf	–
2014	Seo et al. [116]	–	9.3 mm–29.7 mm	–
2014	Zhang et al. [117]	2 mm × 2 mm	4 mm–inf	62,500 mm
2014	Noda et al. [21]	10 mm × 10 mm	82.6 mm–153.6 mm	13,022.84 mm
2015	Nicolas et al. [118]	3 mm × 3 mm	100 mm–inf	337.5 mm
2015	Ashtiani et al. [119]	–	5.8 mm–10.1 mm	–
2015	Ashtiani et al. [120]	–	6.4 mm–33.7 mm	–
2017	Almoallem et al. [121]	1 mm × 1 mm	14 mm–67 mm	609.91 mm
2019	Seo et al. [122]	–	9.3 mm–29.3 mm	–
2022	Xu et al. [123]	0.1 mm × 0.1 mm	0.92 mm–1.42 mm	85.69 mm
2024	Tang et al. [124,125]	1.5 mm × 1.5 mm	31.6 mm–inf	422.48 mm

Tips: In the Size column, the data without any annotations indicates that the shape of the mirror is circular. In the Focal Length Variation column, inf represents the infinite focal length. inf represents the infinite focal length. inf represents the infinite focal length.

**Table 5:** Summary of characteristics of MEMS reflective varifocal mirrors.

	Type	Electrostatic	Piezoelectric	Thermal	Electromagnetic	Fluidic
Characteristics						
Stroke		limited	large	large	large	large
Varifocal speed		medium	fast	slow	slow	slow
Energy consumption		low	low	high	high	—
Structure		simple	medium	complex	complex	simple

**Table 6:** Comparison of three types of MEMS varifocal optical elements.

Type		Basic Principle	Achievable Optical Power Variation Maximum	Achievable Varifocal Speed Maximum	Practical Applications
MEMS reflective varifocal mirrors		Parabolic deformation of reflective surface	100 D	460 kHz	Confocal; OCT; AR/VR; Zoom camera; 3D scanning; Laser process; Head-write heads
MEMS varifocal microlenses	Metalenses	Modulation of optical phase with subwavelength microstructures	10 D	2 kHz	Confocal; OCT; Zoom cameras
	Liquid lenses	Deformation of a liquid droplet	100 D	0.3 kHz	
MEMS phased varifocal mirrors		Modulation of optical phase	—	15 kHz	3D scanning
